# Publications in PubMed on Ebola and the 2014 outbreak

**DOI:** 10.12688/f1000research.6206.2

**Published:** 2015-10-09

**Authors:** Andrea Ballabeni, Andrea Boggio

**Affiliations:** 1 Department of Health Policy and Management, Harvard T.H. Chan School of Public Health, Boston, MA, 02115, USA; 2 Department of History and Social Science, Bryant University, Smithfield, RI, 02917, USA

**Keywords:** Ebola, publications, Pubmed

## Abstract

In this research note we examine the biomedical publication output about Ebola in 2014. We show that the volume of publications has dramatically increased in the past year. In 2014 there have been over 888 publications with ‘ebola’ or ‘ebolavirus’ in the title, approximately 13 times the volume of publication of 2013. The rise reflects an impressive growth starting in the month of August, concomitant with or following the surge in infections, deaths and coverage in news and social media. Though non-research articles have been the major contributors to this growth, there has been a substantial increase in original research articles too, including many papers of basic science. The United States has been the country with the highest number of research articles, followed by Canada and the United Kingdom. We present a comprehensive set of charts and facts that, by describing the volumes and nature of publications in 2014, show how the scientific community has responded to the Ebola outbreak and how it might respond to future similar global threats and media events. This information will assist scholars and policymakers in their efforts to improve scientific research policies with the goal of maximizing both public health and knowledge advancement.

## Introduction

The Ebola outbreak that has affected West Africa for over a year has captured the attention of audiences throughout the world. Much has been said and written about this tragic event in the popular media as well as in scientific journals ^1– 11^. The scientific community has certainly vigorously debated about the Ebola outbreak. However, no study has so far assessed the impact of the current African epidemic on scientific production. In this research note we try to fill this gap by examining the volume and nature of the biomedical publication output about Ebola in 2014. We believe this study is important as it may lead to insights as to how the global scientific and health communities react to these kinds of events. The note looks at general trends in Ebola-related publications, at the type of papers that were published and at the extent to which countries have contributed to the Ebola literature. This information will be useful to researchers and policymakers aspiring to increase the impact of scientists’ work on public health as well as on the pure advancement of knowledge.

## Methods and data

### Searching criteria

**Figure S1.**
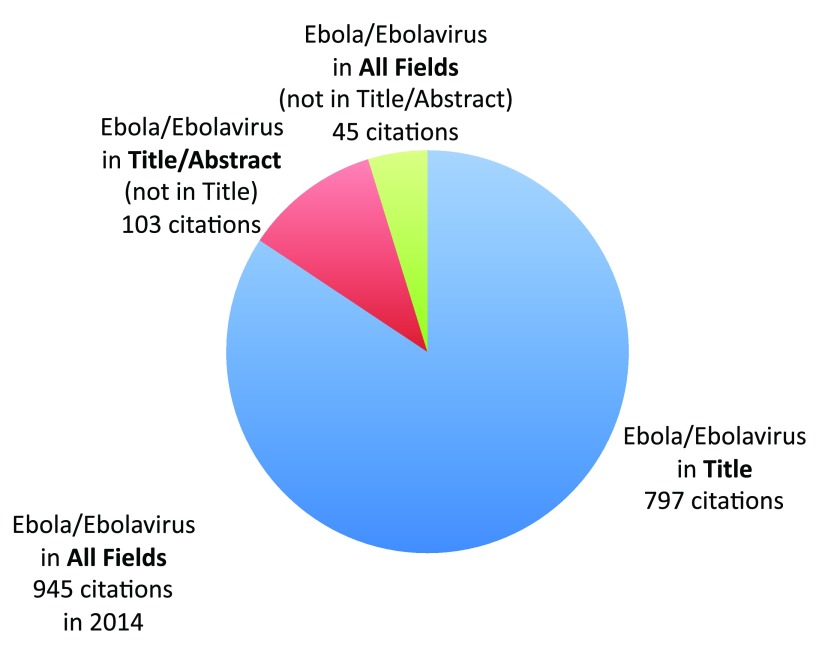
Numbers of 2014 citations with ‘ebola’ or ‘ebolavirus’ in title, title/abstract or all fields.

**Figure S2.**
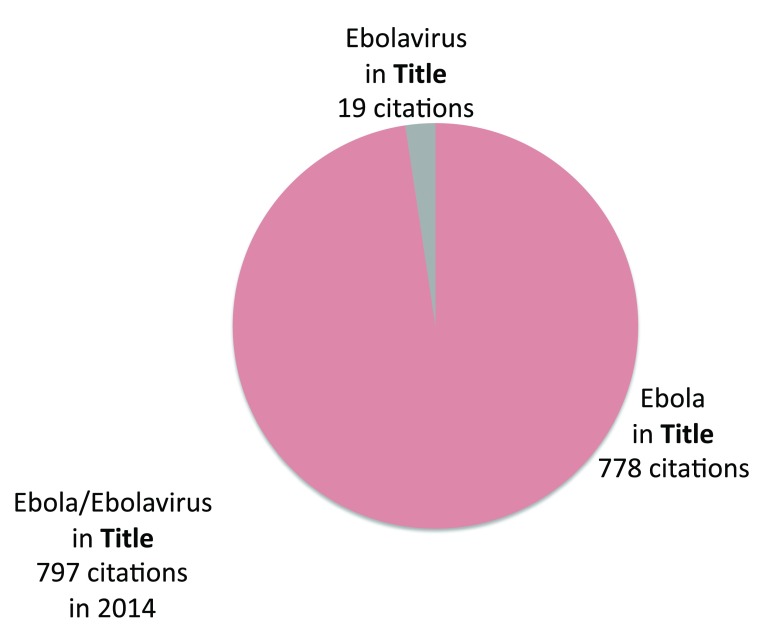
Number of 2014 citations with ‘ebola’ vs ‘ebolavirus’ in title field.

All searches were performed in PubMed ( http://www.ncbi.nlm.nih.gov/pubmed/), a public search engine maintained by the United States National Library of Medicine (NLM) at the National Institutes of Health (NIH) that provides access to over 24 million citations in all fields of life sciences, mostly from the MEDLINE (Medical Literature Analysis and retrieval System Online) database. To calculate the number of publications on Ebola in 2014, we ran a search by using the ‘custom range’ function set to 2014 publications and searched for ‘ebola OR ebolavirus’ in ‘all fields’, in ‘title/abstract’ field or in ‘title’ field. We retrieved, respectively, 945, 900 and 797 citations ( Figure S1) (sheet 1). We also ran the search using the ‘MeSH major topic’ filter and retrieved 945 citations (sheet 1). Upon reviewing the results, we noticed that some citations retrieved when searching only in ‘all fields’ or ‘title/abstract’ or by using the ‘MeSH’ filter were not publications discussing some specific aspect of Ebola; for this reason, we decided to only use the 797 citations retrieved with searches in the title. Throughout the paper we will therefore not specify the search field and use title as the default option. We also searched for ‘ebola’ and ‘ebolavirus’ separately. We retrieved 778 and 19 citations, respectively ( Figure S2) (sheet 1). Given that both ‘ebola’ and ‘ebolavirus’ were used in publications titles, we decided henceforth to use ‘ebola OR ebolavirus’ as the default search term.

Dataset and selected figures of biomedical publications on Ebola in 2014 The database contains data about the biomedical publications on Ebola in 2014.The volumes of publications and the classifications were determined by using the PubMed search engine. The data are ordered according to different criteria. Columns/row headings and embedded comments describe the contents of the columns/rows. Columns A-K of sheets ‘Clinical Trial term’, ‘Canada’, ‘China’, ‘France’, ‘Germany’, ‘Guinea’, ‘Liberia’, ‘Sierra Leone’, ‘UK’, ‘USA’ were from csv files downloaded from PubMed searches. Further information about the methodology and the data is contained in the associated article. All data and figures mentioned in the associated article and this figshare entry are linked to a sheet of this database.
Figure 3 Numbers of citations with ‘ebola’ or ‘ebolavirus’ in title from 1995 to 2014.
Figure 4 Numbers of citations (abstract available) with ‘ebola’ or ‘ebolavirus’ in title from 1995 to 2014.
Figure 5 Proportions of citations (‘ebola’ or ‘ebolavirus’ in title) with abstract available from 2005 to 2014.
Figure 15 Proportions of citations (‘ebola’ or ‘ebolavirus’ in title) with abstract available with the indicated search terms in the title/abstract. Year 2014.
Figure 16 Proportions of citations (‘ebola’ or ‘ebolavirus’ in title) with the search term ‘clinical trial’ in the title/abstract. The number of publications about original clinical trial studies is also indicated. Year 2014.
Figure 17 Numbers of citations (‘ebola’ or ‘ebolavirus’ in title) per month. Year 2014.
Figure 18 Numbers of citations (‘ebola’ or ‘ebolavirus’ in title) with abstract available per month. Year 2014.
Figure 19 Subjective classification for ‘current outbreak’ focus of 2014 citations (‘ebola’ or ‘ebolavirus’ in title). Remaining citations were assigned to one (and only one) of the indicated discipline/area categories. Year 2014.
Figure 20 Subjective classification for current outbreak focus or, alternatively, for the indicated discipline/area categories of 2014 citations (‘ebola’ or ‘ebolavirus’ in title) per month. Citations were assigned to one (and only one) category, similarly to Figure 19. Year 2014.
Figure 21 Numbers of citations (‘ebola’ or ‘ebolavirus’ in title) during 2014 with search term ‘outbreak’ in title/abstract.
Figure 22 Numbers of citations (‘ebola’ or ‘ebolavirus’ in title) during 2014 with search term ‘Africa’ in title/abstract.
Figure 23 Numbers of citations (‘ebola’ or ‘ebolavirus’ in title) during 2014 with search terms ‘vaccine’ or ‘vaccines’ in title/abstract.
Figure 24 Numbers of total biomedical citations of the 20 countries with most total biomedical publications. Year 2014.
Figure 25 Numbers of citations (‘ebola’ or ‘ebolavirus’ in title) of the 20 countries with most total biomedical publications. Year 2014.
Figure 28 Numbers of total citations with abstract available of the 20 countries with most total biomedical publications. Year 2014.
Figure 29 Numbers of citations (‘ebola’ or ‘ebolavirus’ in title) with abstract available of the 20 countries with most total biomedical publications. Year 2014.
Figure 31 Numbers of citations (‘ebola’ or ‘ebolavirus’ in title) of the six countries with most Ebola-related publications. Citations (‘ebola’ or ‘ebolavirus’ in title) with abstract available are also shown. Year 2014.
Figure 32 ‘Manual’ control test of the method for country affiliation attribution. Numbers of citations (‘ebola’ or ‘ebolavirus’ in title) with abstract available of the six countries with most Ebola-related publications. The numbers of publications automatically retrieved or with real ‘any author’, ‘first author’ or ‘last author’ with the proper country affiliation are indicated. 100% of the citations had at least one author (‘any author’) with the proper country affiliation, thus indicating accuracy of the method. Year 2014.
Figure 33 Subjective classification for article type of citations (‘ebola’ or ‘ebolavirus’ in title) with abstract available of the United States and Canada, the two countries with most Ebola-related publications with abstract available. Year 2014.
Figure 34 Subjective classification for discipline/area of citations (‘ebola’ or ‘ebolavirus’ in title) with abstract available of the United States and Canada, the two countries with most Ebola-related publications with abstract available. Citations were assigned only to the more relevant category, except citations related to specific aspects of the 2014 outbreak that were assigned optionally and in addition to the other categories. Year 2014.
Figure 35
Numbers of total biomedical citations and citations (‘ebola’ or ‘ebolavirus’ in title) of Sierra Leone, Liberia and Guinea, the three countries with most Ebola cases. Year 2014. Click here for additional data file. Copyright: © 2015 Ballabeni A and Boggio A 2015 Data associated with the article are available under the terms of the Creative Commons Zero "No rights reserved" data waiver (CC0 1.0 Public domain dedication).

### Ebola publications output 1995–2014

**Figure S3.**
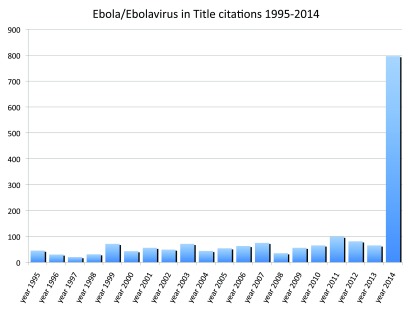
Numbers of citations with ‘ebola’ or ‘ebolavirus’ in title from 1995 to 2014.

To determine recent trends on publications, we looked at the publications record on Ebola for the past 20 years (from 1995 to 2014). Using the ‘custom range’ function, we retrieved a total of 1869 citations. A year-by-year analysis shows that while the publication output did not differ substantially from 1995 to 2013, there was a staggering increase in publication output in 2014 as evidenced by the fact that 42.6% of all the papers published during the time period 1995–2014 were published in the last year alone ( Figure S3) (sheet 2).

**Figure S4.**
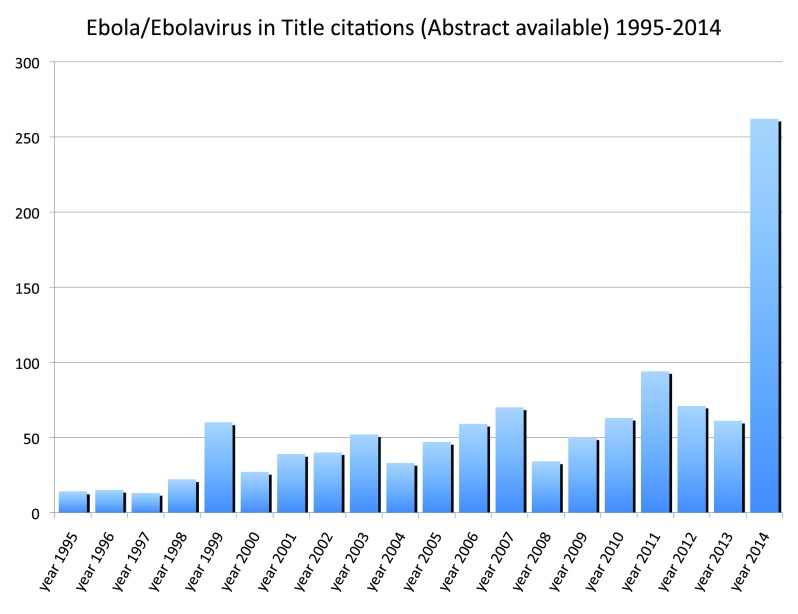
Numbers of citations (abstract available) with ‘ebola’ or ‘ebolavirus’ in title from 1995 to 2014.

**Figure S5.**
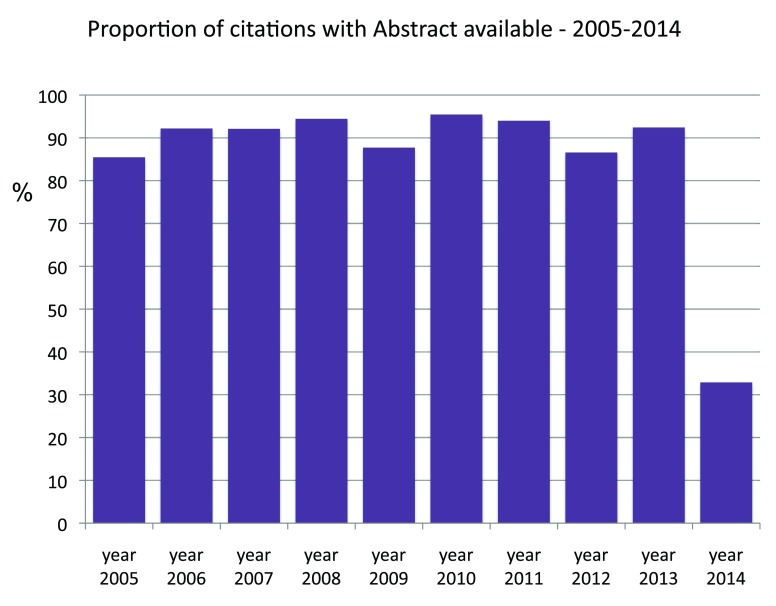
Proportions of citations (‘ebola’ or ‘ebolavirus’ in title) with abstract available from 2005 to 2014.

We then determined the number of publications with an abstract available. As research articles or research notes usually have an abstract but editorials, commentaries, short letters and several other types of publication usually do not, the publications with an abstract better reflect the real output in original research papers. We observed again a remarkable increase in the year 2014, which had 23.3% of all publications of the last 20 years. In particular, the publications with an abstract available in 2014 were almost 5 times the publications with an abstract available in 2013 ( Figure S4) (sheet 2). Interestingly, the proportion of publications with an available abstract in 2014 was considerably lower than in all the previous years: for example, while in 2013 the proportion was 92.4%, in 2014 it was only 32.9% ( Figure S5) (sheet 2). Given that an abstract, when present, is usually made available at the same time the article appears in PubMed, these results indicate that the majority of publications in 2014, differently from the previous years, were non-research papers.

### Analysis by ‘article type’

**Figure S6.**
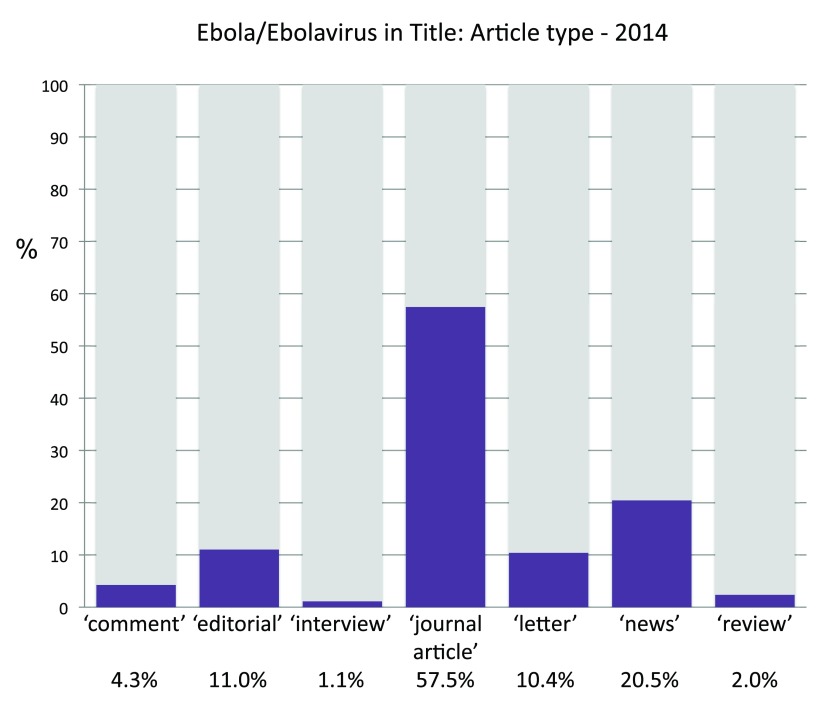
Proportions of citations (‘ebola’ or ‘ebolavirus’ in title) by different PubMed ‘article type’ filters. Year 2014.

**Figure S7.**
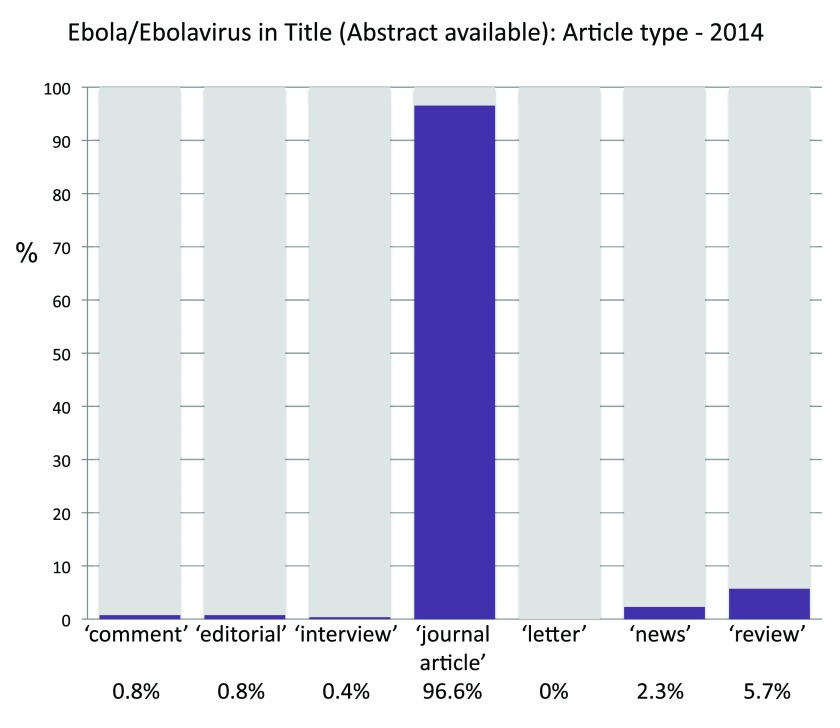
Proportions of citations (‘ebola’ or ‘ebolavirus’ in title) with abstract available by different PubMed ‘article type’ filters. Year 2014.

To determine the article types of 2014 Ebola publications we first used the ‘article types’ filters of PubMed. ‘Comments’ were 4.3%, ‘editorials’ 11.0%, ‘interviews’ 1.1%, ‘journal articles’ 57.5%, ‘letters’ 10.4%, ‘news’ 20.5% and ‘reviews’ 2.0% ( Figure S6) (sheet 3) (note that the sum of percentages is more than 100% because PubMed can assign multiple classifications to the same article). Even if these filters lead to results that may not always be corresponding to the type of items that appears in the database (not all publications are assigned to a PubMed ‘article type’), these numbers indicate the presence of many citations that were not original research articles. By searching for only abstract available citations, we instead obtained very different numbers. ‘Comments’ were 0.8%, ‘editorials’ 0.8%, ‘interviews’ 0.4%, ‘journal articles’ 96.6%, ‘letters’ 0%, ‘news’ 2.3% and ‘reviews’ 5.7% ( Figure S7) (sheet 3). These numbers show, as expected, that the vast majority of publications with an abstract available did not belong to the categories of short articles like editorials but instead belonged to the ‘journal article’ category.

### Analysis by text availability

**Figure S8.**
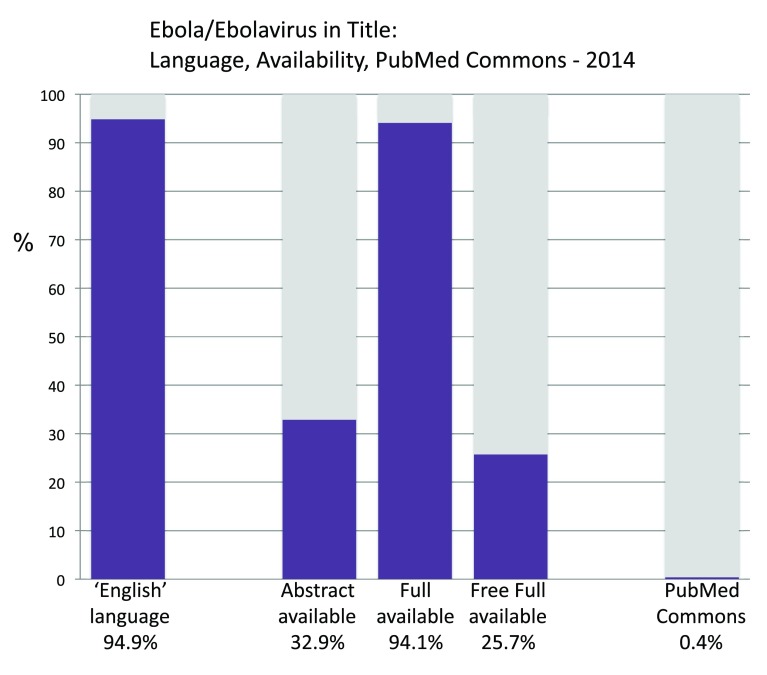
Proportions of citations (‘ebola’ or ‘ebolavirus’ in title) in English, by text availability and by presence of PubMed Commons comments. Year 2014.

**Figure S9.**
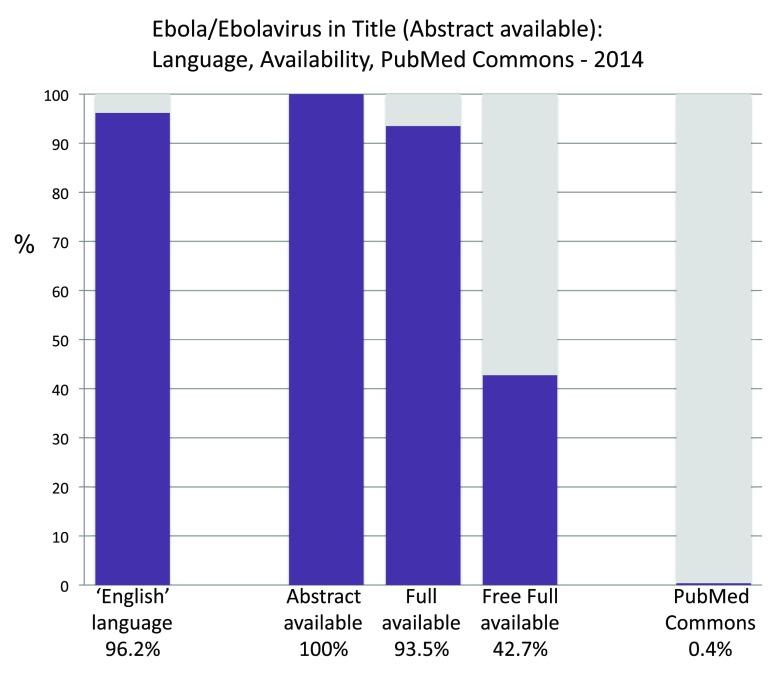
Proportions of citations (‘ebola’ or ‘ebolavirus’ in title) with abstract available in English, by text availability and by presence of PubMed Commons comments. Year 2014.

We then examined three other features: the language, full text availability and the citations in PubMed Commons. By using the English language filter, we found that 94.9% of the publications were written in English. By using the ‘abstract available’, the ‘full text’ and the ‘free full text’ filters we determined that 32.9% of publications had the abstract available, 94.1% had the full text available (either for a fee or at no charge) and 25.7% had the full text available at no charge. Only 0.4% of the publications had comments in PubMed Commons ( Figure S8) (sheet 3). By repeating the same searches with publications with an abstract available, we observed that 96.2% of the articles were written in English, 100% (as obvious) had an abstract available, 93.5% had the full text available (either for a fee or at no charge) and 42.7% had the full text available at no charge. Again only 0.4% had comments in PubMed Commons ( Figure S9) (sheet 3).

**Figure S10.**
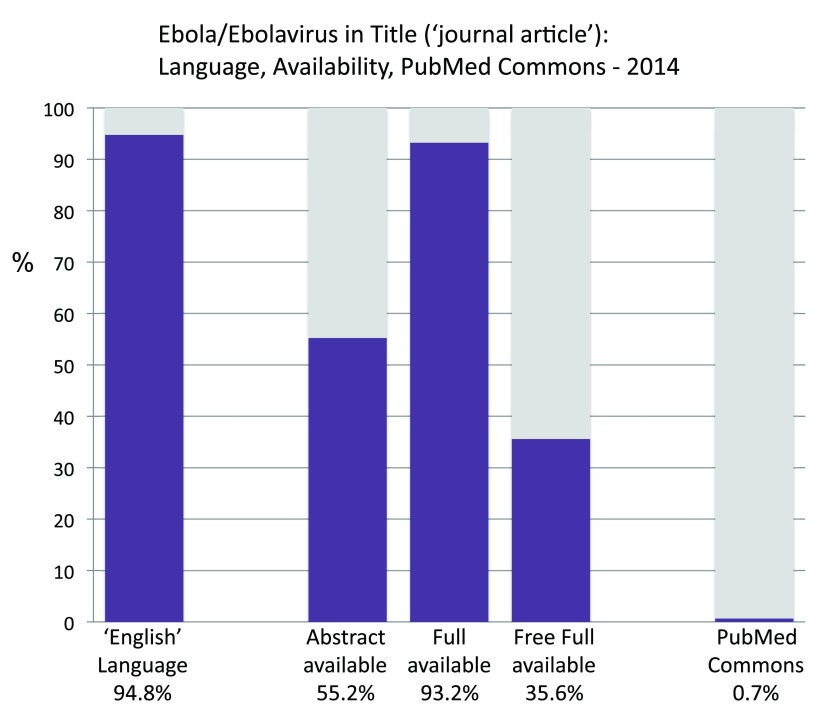
Proportions of ‘article journal’ citations (‘ebola’ or ‘ebolavirus’ in title) in English, by text availability and by presence of PubMed Commons comments. Year 2014.

We also analyzed the language, text availability and PubMed Commons citations only for the ‘journal articles’. 94.8% of these citations were written in English, 55.2% had an abstract available, 93.2% had the full text available (either for a fee or at no charge) and 35.6% had the full text available at no charge. Only 0.7% had comments in PubMed Commons ( Figure S10) (sheet 3).

**Figure S11.**
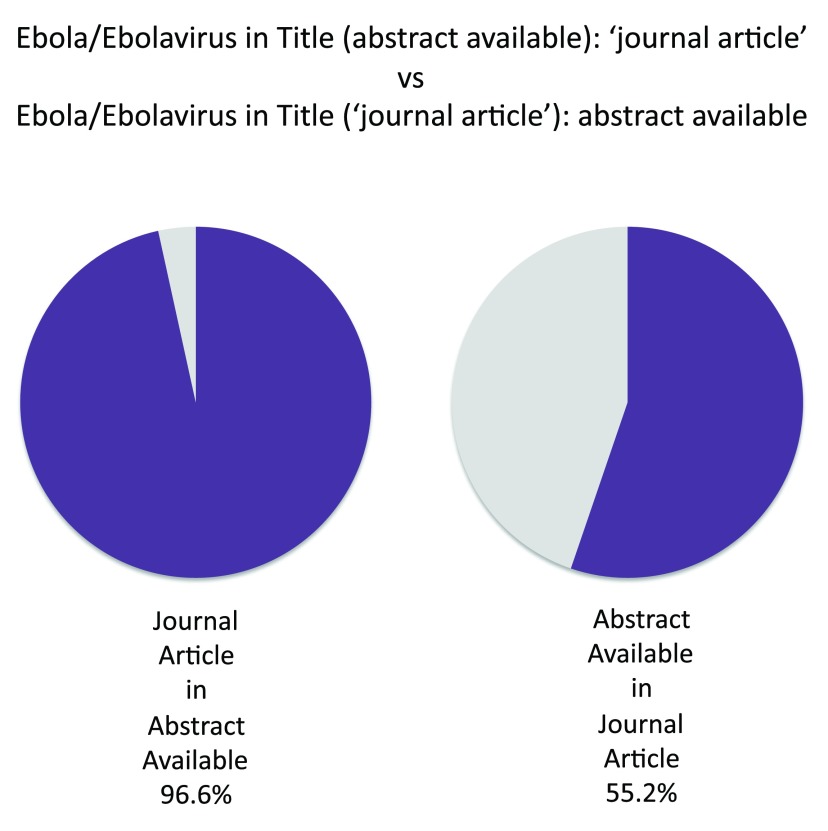
Proportion of citations (‘ebola’ or ‘ebolavirus’ in title) with abstract available that are ‘journal articles’ and proportion of ‘journal article’ citations (‘ebola’ or ‘ebolavirus’ in title) that have an abstract available. Year 2014.

These results show that most of the publications on Ebola in 2014 did not have an abstract and therefore can be safely classified as non-research papers. Moreover they show that, similarly to the case with biomedical publications in general, almost all the citations had a full text available but only in less than half of the cases the text is freely available at present. These data also show that while 96.6% of the publications with an abstract available were ‘journal articles’, only 55.2% of the ‘journal articles’ had an abstract available ( Figure S11) (sheet 3). Based on this evidence we conclude that abstract availability is most likely a better criterion than the ‘journal article’ to estimate which publications are original research papers.

### Focus analysis by use of search terms

**Figure S12.**
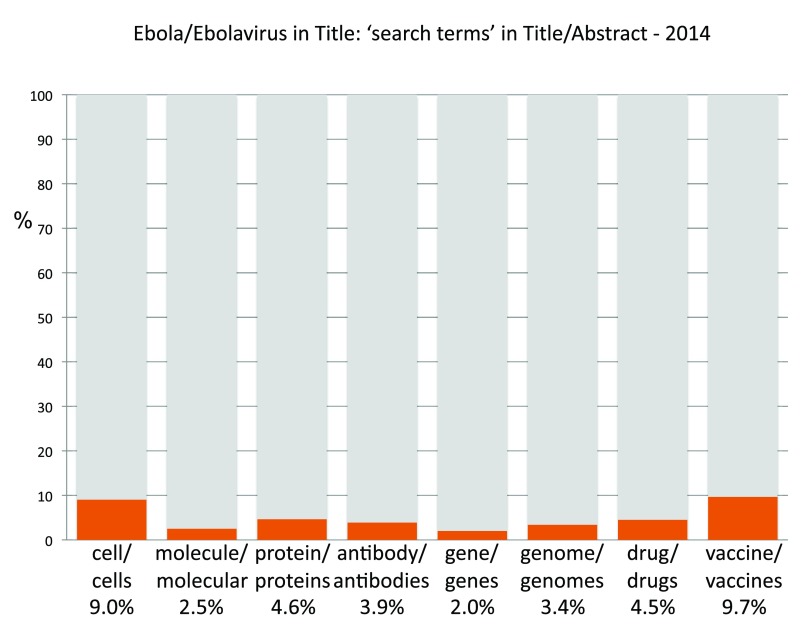
Proportions of citations (‘ebola’ or ‘ebolavirus’ in title) with the indicated search terms in the title/abstract. Year 2014.

**Figure S13.**
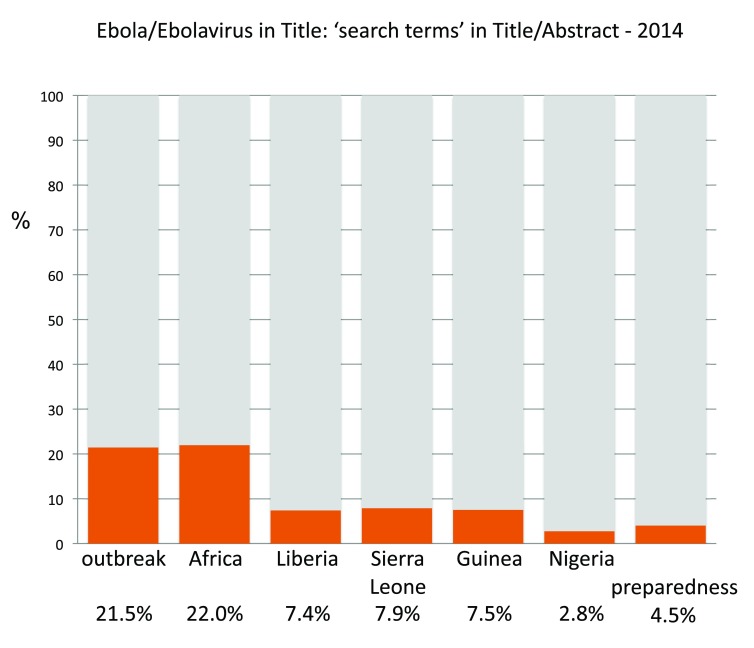
Proportions of citations (‘ebola’ or ‘ebolavirus’ in title) with the indicated search terms in the title/abstract. Year 2014.

To analyze the particular focus of the retrieved papers, we searched for specific terms in the title/abstract field. We first searched for terms related to cell and molecular biology like ‘cell’, ‘molecule’, ‘protein’, ‘antibody’, ‘gene’ and ‘genome’. We also searched for the terms ‘drug’ and ‘vaccine’. All these terms were searched for both the singular and the plural form. The proportions of papers with these terms ranged from 2.0% (for the term ‘gene’) to 9.7% (for the term ‘vaccine’). The term ‘drug’ appeared in 4.5% of the papers ( Figure S12) (sheet 4). We then repeated the analysis with terms more specifically related to the 2014 West Africa outbreak. In particular, we searched for ‘outbreak’, ‘africa’, ‘liberia’, ‘sierra leone’, ‘guinea’, ‘nigeria’, ‘preparedness’. These terms were present in 21.5%, 22.0%, 7.4%, 7.9%, 7.5%, 2.8% and 4.5% of the papers, respectively ( Figure S13) (sheet 4).

**Figure S14.**
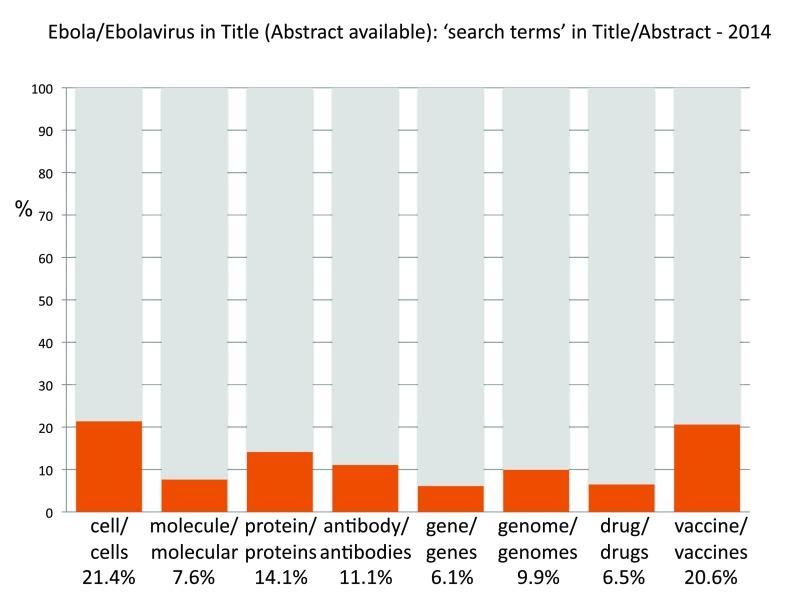
Proportions of citations (‘ebola’ or ‘ebolavirus’ in title) with abstract available with the indicated search terms in the title/abstract. Year 2014.

**Figure S15.**
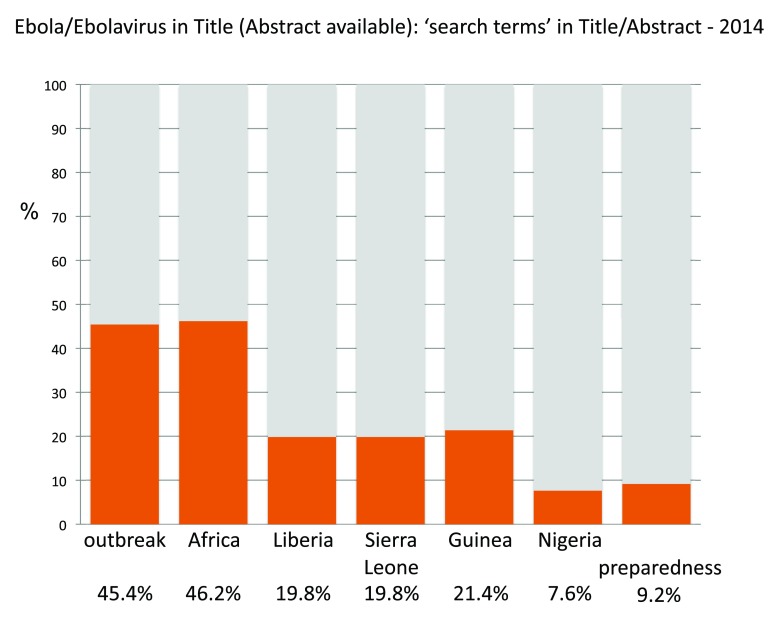
Proportions of citations (‘ebola’ or ‘ebolavirus’ in title) with abstract available with the indicated search terms in the title/abstract. Year 2014.

We then repeated the analyses by taking in consideration only the publications with an abstract available. As expected, the proportions of papers with these terms were higher. The terms ‘drug’ and ‘vaccine’ were in the 6.5% and 20.6% of the publications, respectively ( Figure S14) (sheet 4). The terms ‘outbreak’, ‘africa’, ‘liberia’, ‘sierra leone’, ‘guinea’, ‘nigeria’, ‘preparedness’ were in 45.4%, 46.2%, 19.8%, 19.8%, 21.4%, 7.6% and 9.2% of the publications with an abstract available, respectively ( Figure S15) (sheet 4). As the word Africa was present in almost half of the papers with an abstract available, these results suggest, as expected, that the sharp increase in publications in 2014 was driven mostly by the West Africa outbreak. This was also confirmed by the fact that the years 2012 and 2013 had much lower proportions of papers containing the term ‘Africa’ in the title/abstract (in particular, by performing whole year analyses, we retrieved 9 papers in 2012, 4 in 2013 and 187 in 2014; with regard to publications with an abstract available, 7 papers in 2012, 4 in 2013 and 132 in 2014). Liberia, Sierra Leone and Guinea, the three countries with the most cases of infections, were present in the title or abstract with very similar proportions near to 20% whereas Nigeria, the fourth most affected country but much less affected than the first three, was present in title or abstract of less than 10% of the publications.

### Analysis of clinical trial study publications

**Figure S16.**
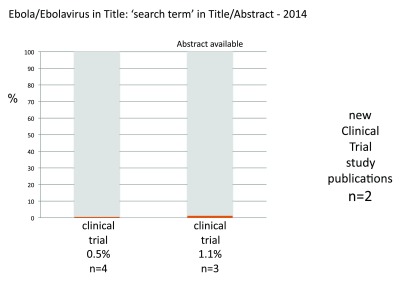
Proportions of citations (‘ebola’ or ‘ebolavirus’ in title) with the search term ‘clinical trial’ in the title/abstract. The number of publications about original clinical trial studies is also indicated. Year 2014.

To obtain the numbers of publications of clinical trials, we used the ‘clinical trial’ filter of PubMed. However, the search led to zero result: no 2014 publication on Ebola was tagged as clinical trial publication. However, this filter of PubMed may not be totally accurate because some clinical trial studies may not be assigned to the ‘clinical trial’ category. Therefore, we also searched for the term ‘clinical trial’ in the title/abstract field. We retrieved 4 publications. Among these, 3 had an abstract available. As the presence of the search term in the title/abstract does not imply that the publication is in fact about a clinical trial, we examined the content of the 4 retrieved publications. We observed that only 2 publications were actually reports of an original clinical trial ( Figure S16) (sheet 4).

### Ebola publications timeline during 2014

**Figure S17.**
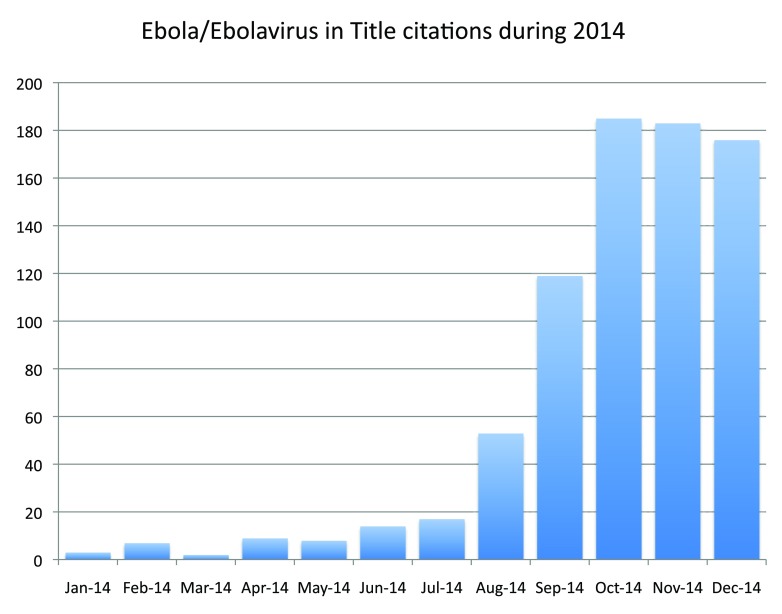
Numbers of citations (‘ebola’ or ‘ebolavirus’ in title) per month. Year 2014.

**Figure S18.**
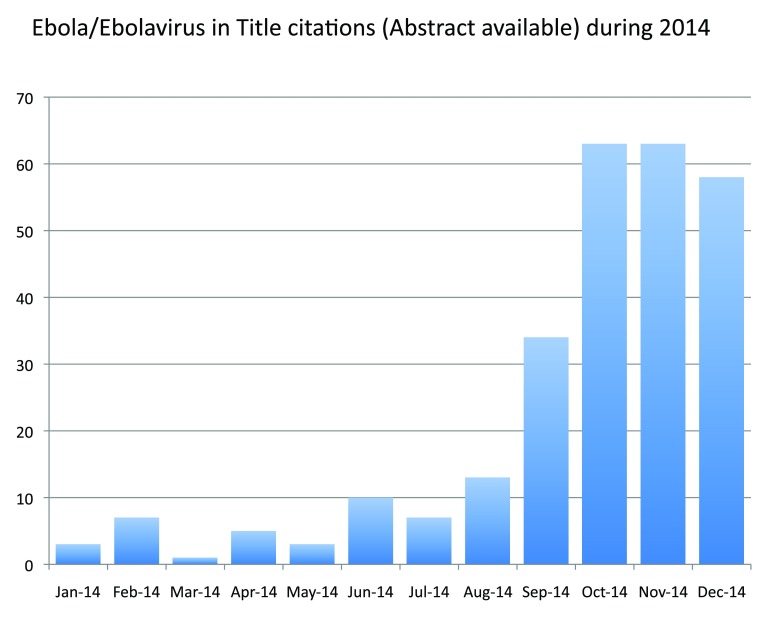
Numbers of citations (‘ebola’ or ‘ebolavirus’ in title) with abstract available per month. Year 2014.

We also analyzed the publication trends within 2014. To this purpose we calculated the volumes of publications for each month of 2014 by using the ‘custom range’ function (for January, we did not include January 1 ^st^ as this would have included a few undetermined publications). In the case of papers published online before the print version, we considered the date of the online publication. As shown in Figure S17 (sheet 5), the vast majority of publications were published after the month of August, with a peak in the month of October and a plateau-like pattern during the last three months of the year. We performed the same analysis also with the publications with an abstract available. We obtained very similar results, with a peak in the months of October and November ( Figure S18) (sheet 5). These results indicate that the increase in publications (both research and non-research papers) only started at the end of the summer of 2014, concomitant with the surge in infection and death cases and more intense reports on part of the mass media.

### Focus analysis by subjective classification

**Figure S19.**
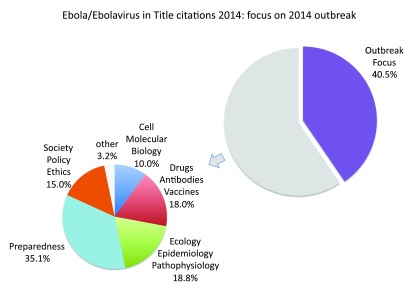
Subjective classification for ‘current outbreak’ focus of 2014 citations (‘ebola’ or ‘ebolavirus’ in title). Remaining citations were assigned to one (and only one) of the indicated discipline/area categories. Year 2014.

**Figure S20.**
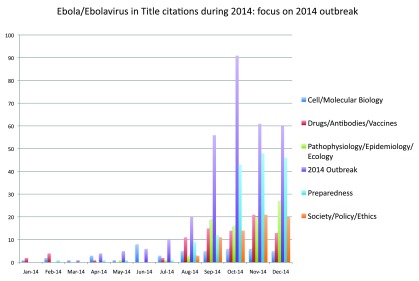
Subjective classification for current outbreak focus or, alternatively, for the indicated discipline/area categories of 2014 citations (‘ebola’ or ‘ebolavirus’ in title) per month. Citations were assigned to one (and only one) category, similarly to Figure S19. Year 2014.

We further investigated the focus of the 2014 publication output by performing a subject-matter analysis. By looking at the title and the abstract, we determined the proportion of papers that had a relevant focus towards one or more aspects of the current 2014 outbreak. We found out that a high proportion (40.5%) of all citations had focused on some aspects of the West Africa epidemic ( Figure S19) (sheet 5). Most of these publications were reports of the epidemic or articles about preparedness or the international response to the crisis. With regard to the other publications, we assigned them to one of the following categories: ‘cell/molecular biology’, ‘drugs/antibodies/vaccines’, ‘pathophysiology/epidemiology/ecology’, ‘preparedness’, ‘society/policy/ethics’ or ‘other’. Even if many papers could be assigned to more than one category, each paper was assigned only to the most applicable category. All papers focusing on drugs/antibodies/vaccines were assigned to the ‘drugs/antibodies/vaccines’ even if many of these could belong also to the ‘cell/molecular biology’ category. We observed that the category with more hits was ‘preparedness’ with 35.1% of the papers, followed by ‘pathophysiology/epidemiology/ecology’ (18.8%), ‘drugs/antibodies/vaccines’ (18.0%), ‘society/policy/ethics’ (15.0%), ‘cell/molecular biology’ (10.0%) and ‘other’ (3.2%) ( Figure S19) (sheet 5). We also analyzed the distribution of these categories on a monthly basis; we observed that the categories ‘outbreak’ and ‘preparedness’ increased substantially after August, with ‘outbreak’ peaking in October and ‘preparedness’ peaking in November. The other categories also increased considerably after August. The category with the smallest growth was ‘cell/molecular biology’; this is not surprising as this is the category that is the least directly connected to the current Africa events ( Figure S20) (sheet 5).

**Figure S21.**
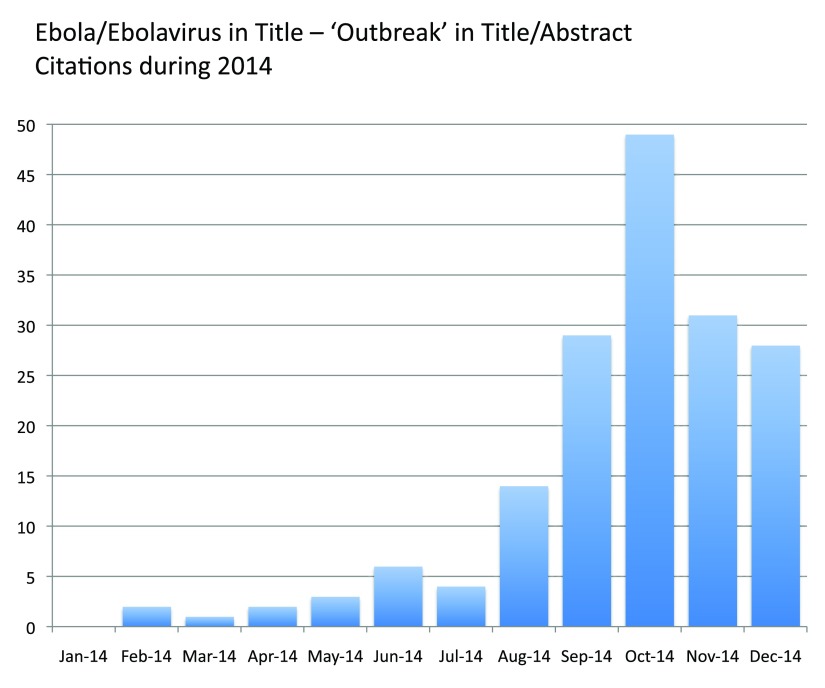
Numbers of citations (‘ebola’ or ‘ebolavirus’ in title) during 2014 with search term ‘ outbreak’ in title/abstract.

**Figure S23.**
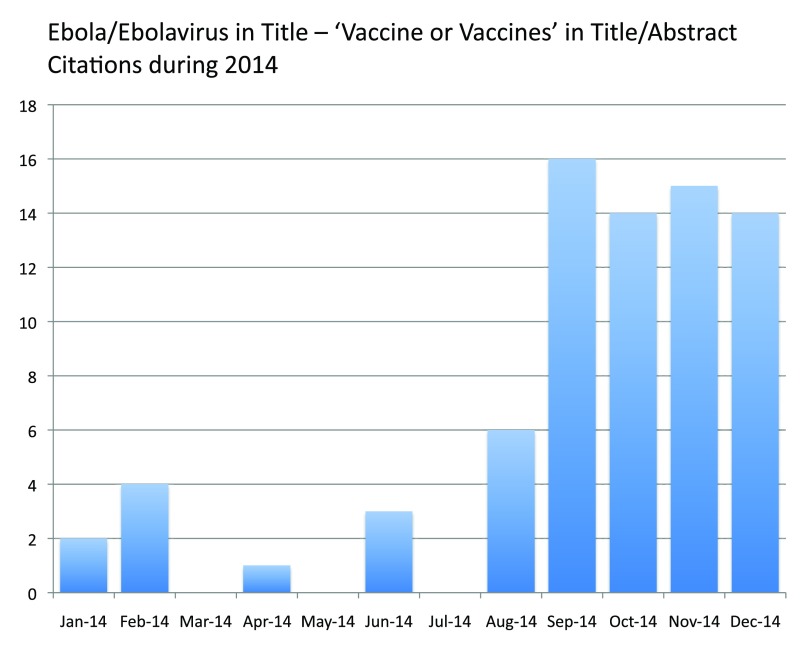
Numbers of citations (‘ebola’ or ‘ebolavirus’ in title) during 2014 with search terms ‘ vaccine’ or ‘ vaccines’ in title/abstract.

In order to corroborate this classification by subject-matter, we also used objective parameters by examining the monthly distribution of papers containing specific terms. We analyzed the monthly distribution of papers with the terms ‘outbreak’, ‘africa’ or ‘vaccine’ in the title/abstract (see Figure S12– Figure S15) (sheet 4) and found that the distribution patterns of papers for ‘outbreak’, ‘africa’ and ‘vaccine’ were similar to the patterns obtained with the subject-matter and subjective classification. In particular, the papers with the term ‘outbreak’ peaked in October and had a distribution very similar to the subject-matter category ( Figure S21– Figure S23) (sheet 5). These results show that the majority of papers published in 2014 were either about the West Africa epidemic or about general preparedness topics. Nonetheless, all discipline/area categories substantially increased during the last part of the year, including papers of basic cell and molecular biology research.

### Analysis of publications by country

**Figure S24.**
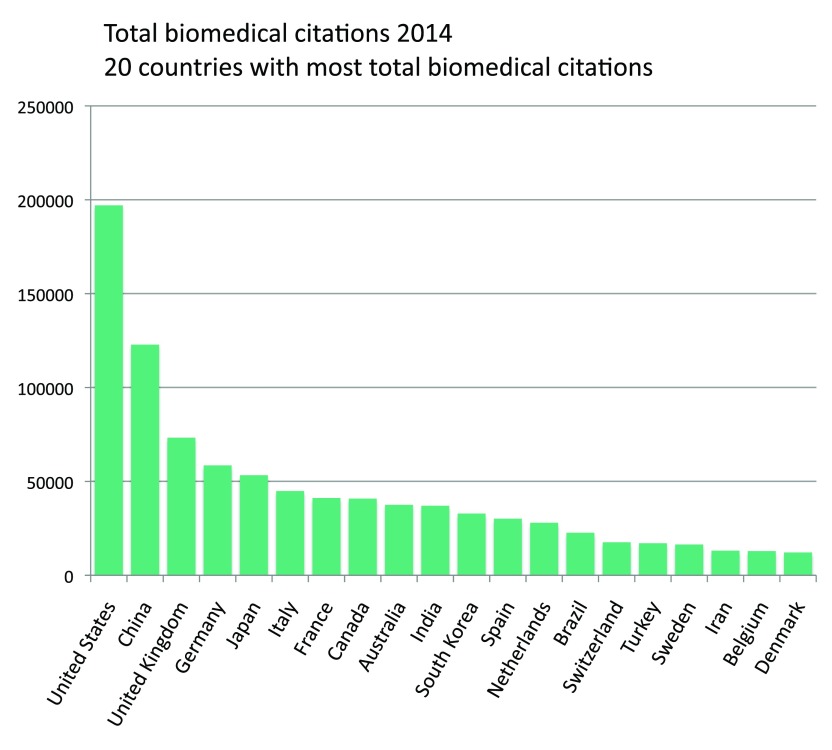
Numbers of total biomedical citations of the 20 countries with most total biomedical publications. Year 2014.

**Figure S25.**
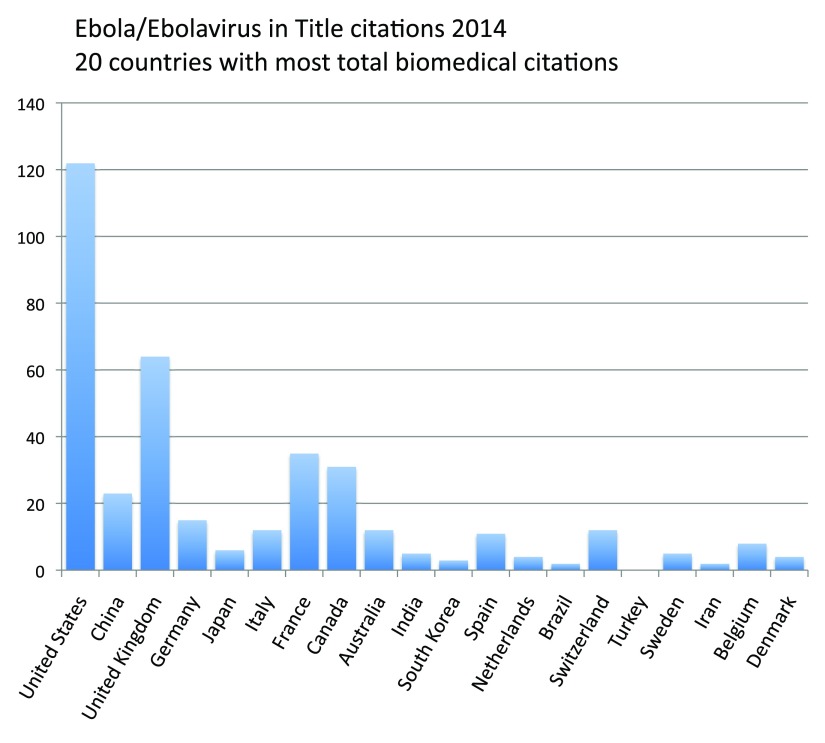
Numbers of citations (‘ebola’ or ‘ebolavirus’ in title) of the 20 countries with most total biomedical publications. Year 2014.

**Figure S26.**
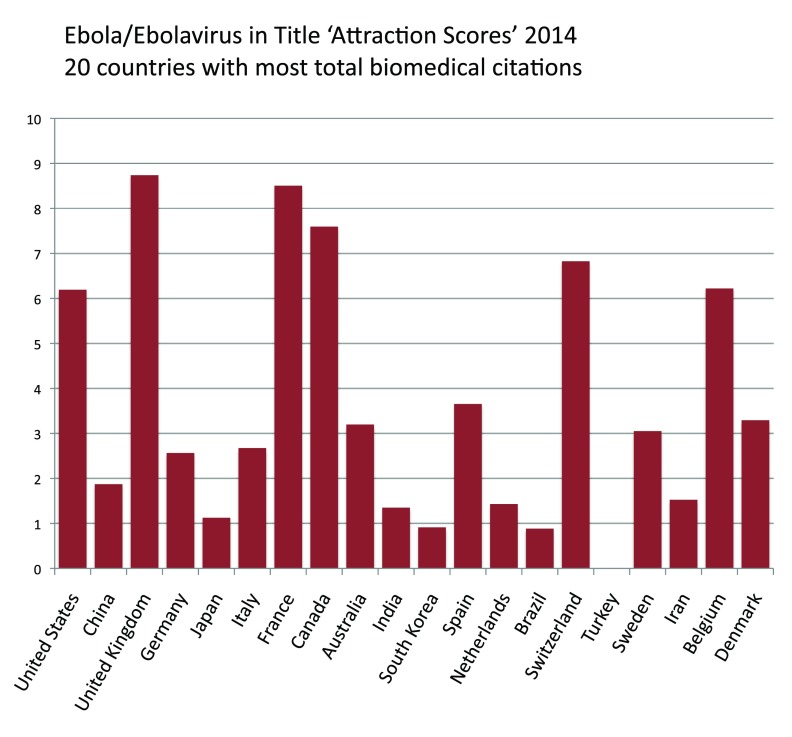
‘Attraction scores’ for citations (‘ebola’ or ‘ebolavirus’ in title) of the 20 countries with most total biomedical publications. ‘Attraction scores’ were calculated by dividing the number of Ebola-related citations by the total biomedical citations of the country and by multiplying by 10,000. Year 2014.

We analyzed the Ebola publications by country. To this end, we used a previously used method ^12^ that relies on the affiliation field provided by PubMed to determine the affiliation country of authors. Before 2013 this method could be used to determine the affiliation country based on the first author only. However, starting from 2014, PubMed provides for most of citations information about every author. Thus, in 2014 this method could be used to quantify the number of papers with at least one author (any author, not only the first one) with a reported affiliation in a given country ^12^. Using this method, we calculated the Ebola publications for the 20 countries with most publications ^12^ (the United States was the country with most total biomedical publications, with almost 20,000 citations in the year 2014, followed by China and the United Kingdom ( Figure S24) (sheet 6)). The United States was the country with by far the most Ebola publications (122 citations), followed by the United Kingdom (64), France (35) and Canada (31) ( Figure 25) (sheet 6). We also calculated the ‘attraction scores’ for the same 20 countries. These scores are a measure of the relative focus towards a topic/field and are calculated by dividing the topic/field-relevant papers by the total number of biomedical papers for the same country (and multiplying by 10,000) ^12^. The ‘attractions scores’ of the 20 countries were very different, as expected by the different patterns of total and Ebola only papers ( Figure S24, Figure S25) (sheet 6). The country with the highest ‘attraction score’ was the United Kingdom, followed by France, Canada and Switzerland ( Figure S26) (sheet 6).

**Figure S27.**
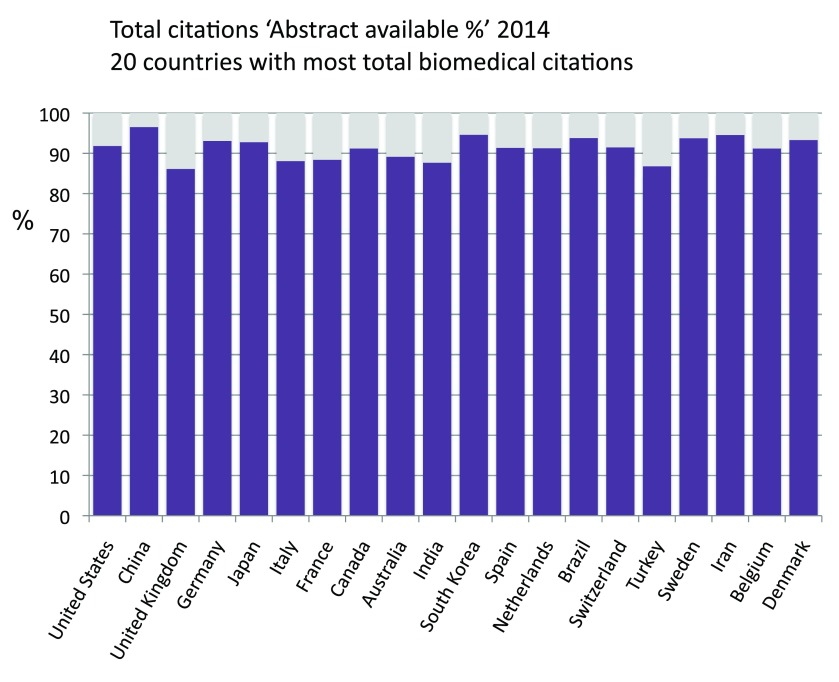
Proportions of total citations with abstract available of the 20 countries with most total biomedical publications. Year 2014.

We then repeated the same analyses by taking in consideration only the papers with an abstract available. If one looks at all biomedical publications, the proportion of publications with an abstract available was very high (on average 91.3%), with very little variability (relative standard deviation was 3.1%) ( Figure S27) (sheet 6). If compared to Ebola-only papers, this number is very high. In fact, only 32.9% of the Ebola papers contained an abstract (see Figure S8) (sheet 3). This shows that, in 2014, the proportions of publications types on Ebola were different from those of the average biomedical publication.

**Figure S28.**
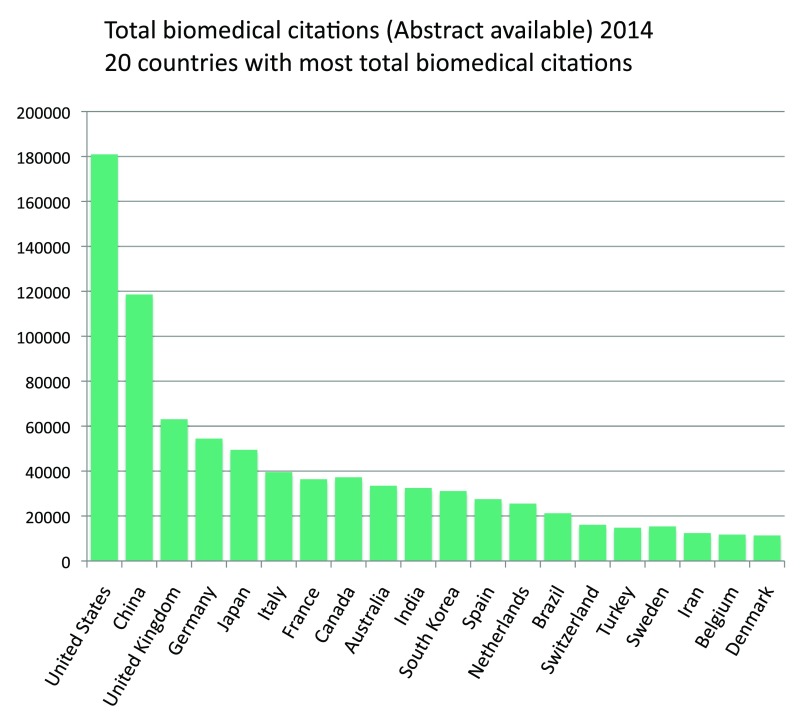
Numbers of total citations with abstract available of the 20 countries with most total biomedical publications. Year 2014.

**Figure S29.**
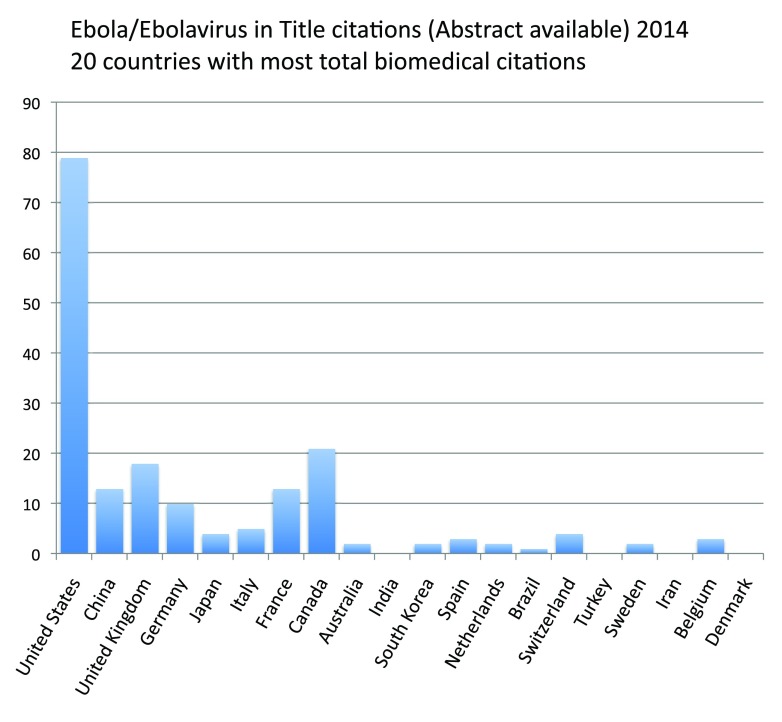
Numbers of citations (‘ebola’ or ‘ebolavirus’ in title) with abstract available of the 20 countries with most total biomedical publications. Year 2014.

**Figure S30.**
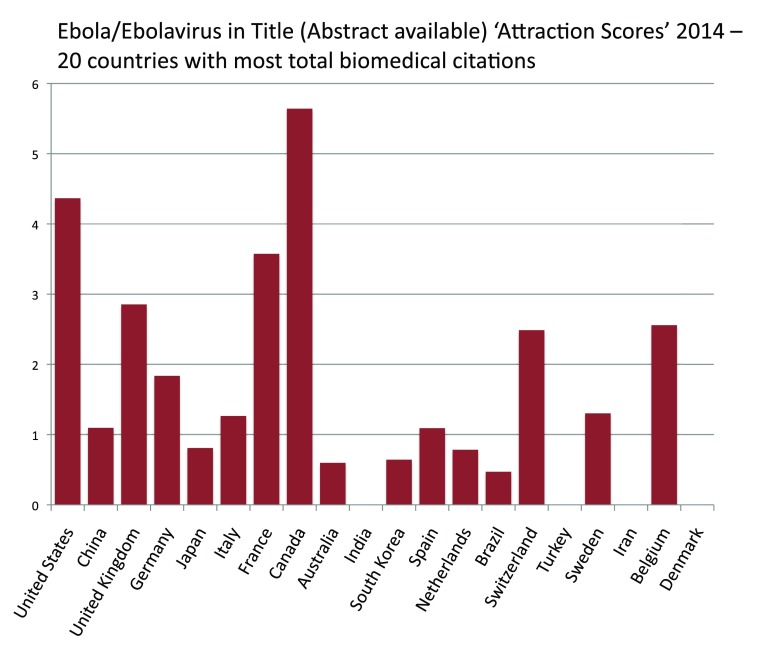
‘Attraction scores’ for citations (‘ebola’ or ‘ebolavirus’ in title) with abstract available of the 20 countries with most total biomedical publications. ‘Attraction scores’ were calculated by dividing the number of Ebola-related citations by the total biomedical citations of the country and by multiplying by 10,000. Year 2014.

We then looked at the only publications with an abstract available (the United States was the country with most total biomedical publications, with over 180,000 citations, followed by China, the United Kingdom and Germany ( Figure S28) (sheet 6). The proportion of Ebola publications attributed to the United States (79 citations) was even higher than in the case of all types of publications, followed by Canada (21) and the United Kingdom (18) ( Figure S29) (sheet 6). Canada was in this case the country with the highest ‘attraction score’, followed by the United States and France ( Figure S30) (sheet 6).

**Figure S31.**
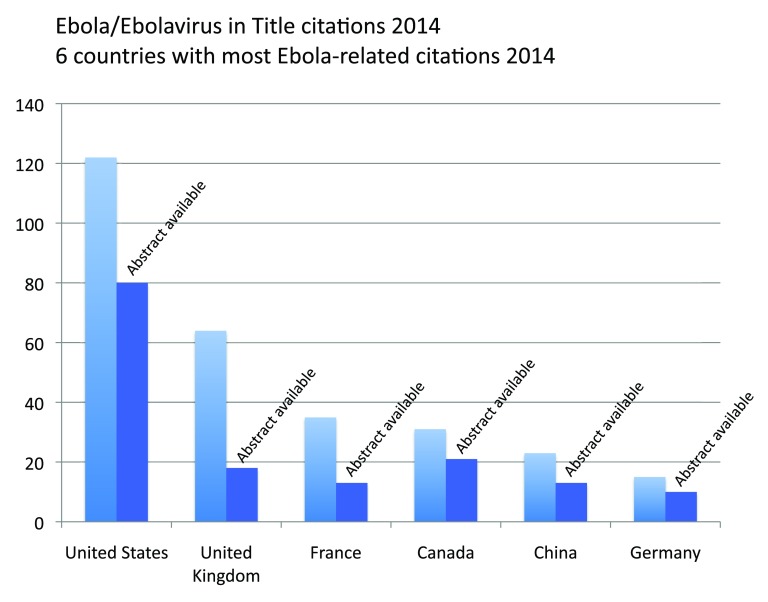
Numbers of citations (‘ebola’ or ‘ebolavirus’ in title) of the six countries with most Ebola-related publications. Citations (‘ebola’ or ‘ebolavirus’ in title) with abstract available are also shown. Year 2014.

**Figure S32.**
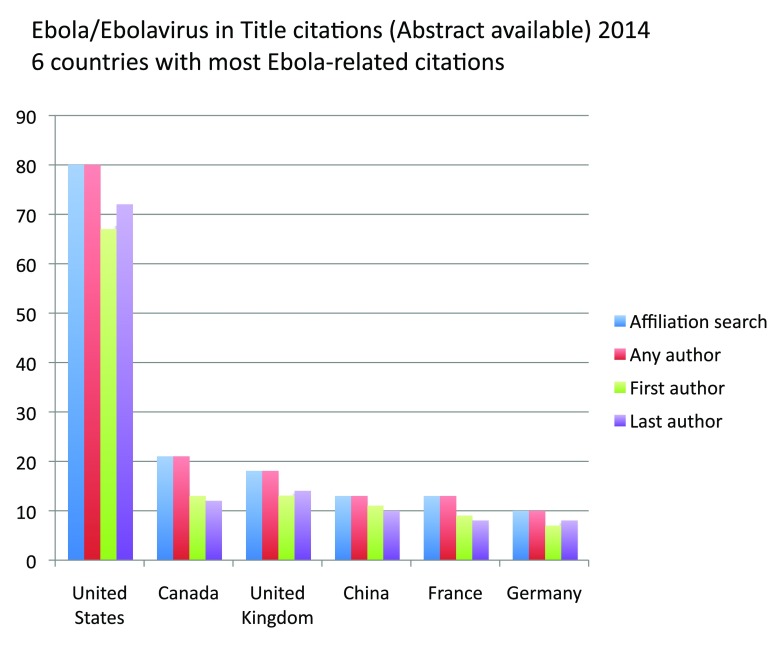
‘Manual’ control test of the method for country affiliation attribution. Numbers of citations (‘ebola’ or ‘ebolavirus’ in title) with abstract available of the six countries with most Ebola-related publications. The numbers of publications automatically retrieved or with real ‘any author’, ‘first author’ or ‘last author’ with the proper country affiliation are indicated. 100% of the citations had at least one author (‘any author’) with the proper country affiliation, thus indicating accuracy of the method. Year 2014.

We finally assessed the accuracy of our method by scrutinizing the six countries with most Ebola publications ( Figure S31) (sheet 7). We looked at the affiliation information of all the papers with an abstract available published by these countries to determine if any author from those countries was indeed present. As a matter of fact, 100% of the 155 analyzed papers had at least one author with at least one affiliation from the searched country; this result proved the accuracy of the method. We then determined the numbers of first and last authors with an affiliation in the searched country. With regard to the first author, we observed that in 73.6% of the papers she/he was from the searched country (China had the largest proportion (84.6%) and Canada the smallest proportion (61.9%)). With regard to the last author, we observed that in 73.9% of the papers she/he was from the searched country (the United States had the largest proportion (90.0%) and Canada had the smallest proportion (57.1%)) ( Figure S32) (sheet 7) (see also USA, UK, France, Canada, China, Germany sheets).

### A closer look at the focus of publications from the United States and Canada

**Figure S33.**
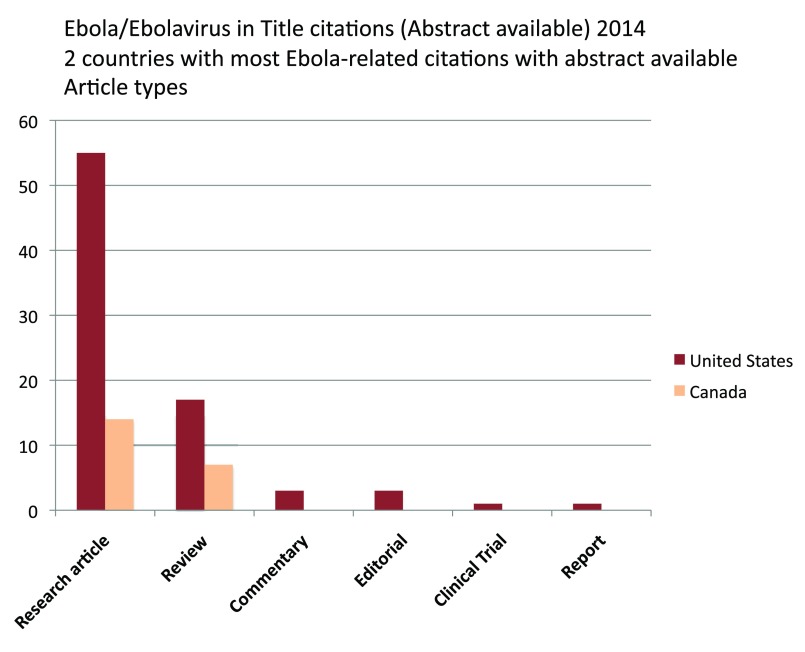
Subjective classification for article type of citations (‘ebola’ or ‘ebolavirus’ in title) with abstract available of the United States and Canada, the two countries with most Ebola-related publications with abstract available. Year 2014.

We took a closer look at the 101 papers with an abstract available published by the United States and Canada - the two countries with most citations with an abstract available. We first examined the article types and found that the vast majority of papers with an abstract available from the United States or Canada were research papers (average 68.3%). Reviews were the second category (average 23.8%). The sum of all the other categories (like commentaries and editorials) was less than 8% of the papers with an abstract available ( Figure S33) (sheet 8). These results indicate that the vast majority of papers with an abstract available were indeed research publications; also, they reveal that the quantity of reviews was higher than the one determined by the ‘review’ filter managed by PubMed.

**Figure S34.**
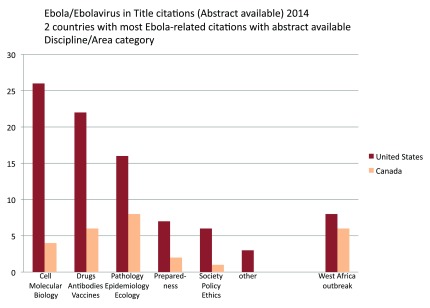
Subjective classification for discipline/area of citations (‘ebola’ or ‘ebolavirus’ in title) with abstract available of the United States and Canada, the two countries with most Ebola-related publications with abstract available. Citations were assigned only to the more relevant category, except citations related to specific aspects of the 2014 outbreak that were assigned optionally and in addition to the other categories. Year 2014.

We then examined the area/field of the same set of 101 publications. As before, we assigned papers to different categories based on the discipline/area of study focus. We observed that 29.7% were about basic ‘cell/molecular biology’, 27.7% about ‘drugs/antibodies/vaccines’, 23.8% about ‘pathophysiology/epidemiology/ecology’, 8.9% about ‘preparedness’, 6.9% about ‘society/policy/ethics’ and 3.0% about ‘other’ areas of study. We also looked at how many of these papers were specifically focused on specific aspects of the current West Africa outbreak. We observed that 13.9% of the papers dealt with some features of the 2014 epidemic. In this case, differently from before, the papers were (optionally) assigned to the ‘outbreak’ category in addition to the assignment to the discipline/area categories ( Figure S34) (sheet 8). The pattern of discipline/area focus of the papers were different between the two countries, with the United States pattern more similar to the average pattern, as also anticipated by the much higher share of publications published by the United States. These results indicate that, differently from the papers without abstracts (e.g. editorials, commentaries, short letters), research papers and extensive reviews focus much more on basic cell and molecular biology or on drugs and vaccines and less on preparedness or on specific aspects of the current epidemic (see also USA and Canada sheets).

### A look at the Ebola literature from the three countries most affected by the 2014 epidemic

**Figure S35.**
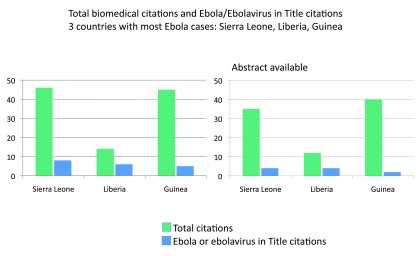
Numbers of total biomedical citations and citations (‘ebola’ or ‘ebolavirus’ in title) of Sierra Leone, Liberia and Guinea, the three countries with most Ebola cases. Year 2014.

**Figure S36.**
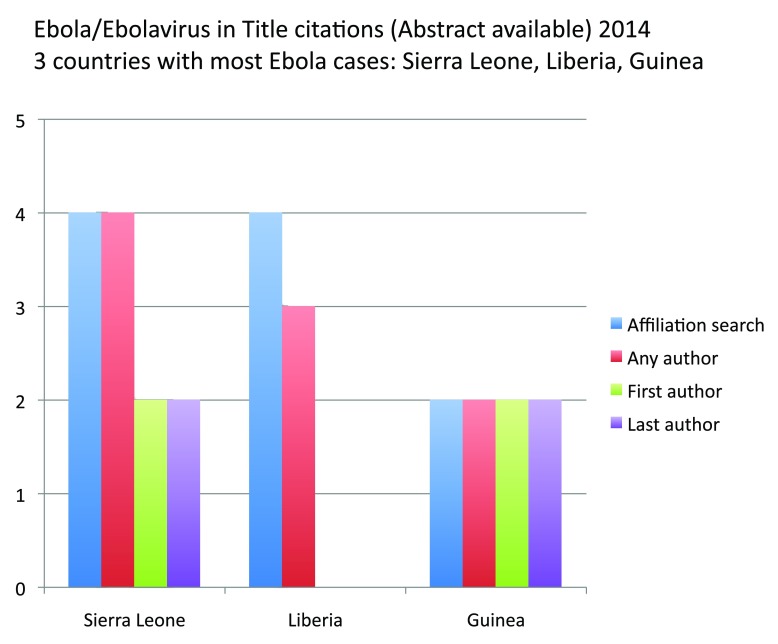
‘Manual’ control test of the method for country affiliation attribution. Numbers of citations (‘ebola’ or ‘ebolavirus’ in title) with abstract available of the three countries with most Ebola cases. The numbers of publications automatically retrieved or with real ‘any author’, ‘first author’ or ‘last author’ with the proper country affiliation are indicated. Year 2014.

We then looked at the publication output of the three countries (Sierra Leone, Liberia and Guinea) that have been most affected by the epidemic, both in terms of cases and deaths ( http://www.cdc.gov/vhf/ebola/outbreaks/2014-west-africa/case-counts.html). We therefore calculated the total numbers of biomedical papers as well as the only papers on Ebola, taking also in consideration only publications with an abstract available. These countries publish very low numbers of biomedical papers: in 2014, 46 by Sierra Leone, 14 by Liberia and 45 by Guinea. All three countries had a high proportion of papers with an abstract available similarly to the 20 countries with most publications (the average was 83.6%). Despite the very small numbers of total biomedical publications, all the three countries published papers on Ebola. Sierra Leone had 4 publications with an abstract available, Liberia 4 and Guinea 2 ( Figure S35) (sheet 9). As expected, the Ebola ‘attraction scores’ for these three countries were very high (sheet 9). As done before, we also controlled for the accuracy of our method for the 10 papers with an abstract available retrieved by searching the affiliation field. Nine of these papers (90% of cases) were authored by at least one person with (at least) an affiliation in one of three countries. This confirmed again the accuracy of our method as only 1 out of total 165 tested papers (so 0.6% of the tested papers) did not have any author with an affiliation truly corresponding to the searched country. We also determined the numbers of first authors and last authors that had a reported affiliation in the searched country. While there were no publications with the first or the last author with a Liberia affiliation, there were 2 publications with the first author having a Sierra Leone affiliation, 2 publications with the first author having a Guinea affiliation, 2 publications with the last author having a Sierra Leone affiliation and 2 publications with the last author having a Guinea affiliation ( Figure S36) (sheet 9). Among the 9 papers with at least one author from one of these three countries, 7 focused on some aspects of the 2014 epidemic. There were also 5 original research articles but 4 of these had neither the first nor the last author from one of the three countries. The only research article with either the first or last author with an affiliation in one of the three countries was a paper from Guinea of the category ‘pathophysiology/epidemiology/ecology’ (see also Sierra Leone, Liberia and Guinea sheets).

### Tendency to mention ‘ebola OR ebolavirus’ in the title in years 2013 and 2014 (searches updated in September 2015)

**Figure S37.**
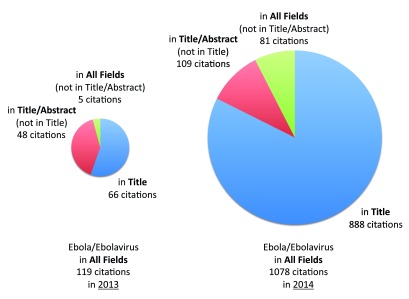
Numbers of 2013 and 2014 citations with ‘ebola’ or ‘ebolavirus’ in title, title/abstract or all fields. Pie chart sizes of 2013 and 2014 are in proportion according to the respective numbers of citations. Searches were made in September 2015 (‘Update September 2015’ sheet).

In September 2015 we repeated the searches for citations of 2014 with ‘ebola OR ebolavirus’ in different fields. We noticed a slight increase in the number of citations in comparison to the previous searches. For example, whereas in the February searches we retrieved 797 citations with ‘ebola OR ebolavirus’ in the title field, in the September searches we retrieved 888 citations (11.1% increase). This was expected considering that some citations could be retrieved by PubMed only a few months after publication. The proportions of citations with ‘ebola OR ebolavirus’ in different fields were similar between the searches made in February and the searches made in September. We also performed the same searches for 2013. Interestingly, we noticed a significant difference with regard to the proportions of ‘ebola OR ebolavirus’ in different fields in the two years. In particular, the proportion of publications with ‘ebola OR ebolavirus’ in the title field was in 2014 much higher than in 2013 (82.4% in 2014 vs 55.5% in 2013) ( Figure S37).

## Conclusions

In this report we have analyzed the volumes and characteristics of the publications about Ebola virus during the year 2014. These are some of the key findings:

1. There were 900 (updated 997) citations with the terms ‘Ebola’ or ‘Ebolavirus’ in the title/abstract, including 797 (updated 888) citations with one of these terms in the title.
2. The volume of citations with ‘ebola OR ebolavirus’ in the title was over 12-fold (updated 13-fold) the volume of citations of 2013. The difference for citations with ‘ebola OR ebolavirus’ in any field was instead only 9-fold. This was due to the much higher proportion of citations with ‘ebola OR ebolavirus’ in the title in 2014 (82.4% in 2014 vs 55.5% in 2013).
3. The volume of citations with an abstract available was over 4-fold the volume of citations of 2013.
4. The proportion of papers with an abstract available was 32.9% whereas in 2013 it was 92.4%. The proportion for all biomedical papers published by the top 20 publishing countries was 91.3%.
5. 20.6% of the papers with an abstract available contained the terms ‘vaccine’ or vaccines’ in the title/abstract.
6. 46.2% of the papers with an abstract available contained the term ‘Africa’ in the title/abstract.
7. There were only 2 publications about an original clinical trial study.
8. 85.4% of the papers were published in the last 4 months of the year.
9. 81.6% of the papers with an abstract available were published in the last 4 months of the year.
10. The peak of papers with the term ‘outbreak’ in the title/abstract was in October.
11. The country with most publications on Ebola was the United States with 122 citations, followed by the United Kingdom with 64 citations.
12. The country with most publications on Ebola with an abstract available was the United States with 80 citations, followed by Canada with 21 citations.
13. Among the 80 publications with an abstract available and with at least one author affiliated to a United States institution, the first and last authors with an affiliation in this country were 67 and 72, respectively.
14. Among publications with an abstract available and with either United States or Canada affiliations, 68.3% were original research papers.
15. Among publications with an abstract available and with either United States or Canada affiliations, 27.7% were studies about ‘drugs/antibodies/vaccines’ and 29.7% were other types of ‘cell/molecular biology’ studies.
16. There were 9 publications with an abstract available with at least one author with an affiliation in Sierra Leone, Liberia or Guinea, including 4 with either the first or the last author with an affiliation in these countries. There were also 5 original research papers with at least one author from these countries.

The data of this report show a very dramatic change in the volume and nature of Ebola publications in 2014. While non-research papers have played a major role in the staggering increase in publications, there has also been a dramatic increase of original research articles or research notes (like this same research note), including many studies on potential new drugs or vaccines or related to basic biological research. Interestingly, the increase in publications took place in the second part of 2014 although the epidemic had already started at the very end of 2013. The surge in publications (research and non-research papers) was therefore concomitant with the surge in number of cases and deaths and thus certainly due to the current West Africa outbreak. Even if an analysis of the causes is beyond the scope of this note, this surge could be due to several reasons: a genuine increased interest towards a major global health threat, a perception of easiness in publishing by writing about Ebola or by simply inserting a few comments on Ebola in the title/abstract, a strategic move in anticipation of increased funding for the Ebola field and several other reasons. In any case, it has to be taken in consideration that articles’ submissions and publications dates are never simultaneous; in addition, this time interval often varies between research articles and commentary pieces. Moreover, while commentary pieces usually involve only writing and submission of the paper, research papers always require experimental work before writing. Even if an in-depth evaluation of these factors is beyond the purposes of this research note, we believe that it can be safely argued that the findings of this research note indicate that the scientific community started publishing many more editorials, letters and comments (and some of the research articles) on Ebola only during or after the massive increase in deaths and media coverage. Even if the rises in scientific papers and media coverage could be independent, it can be speculated that at least a fraction of them were in fact inter-dependent. In particular, it is plausible that many scientific publications were conceived and published as a consequence of the extensive coverage in news and social media.

Given the breadth and accuracy of PubMed-searched databases we believe the data presented in this paper are a good representation of the publication volumes. However, we believe that it will be worth confirming these findings in the future by using other database such as Scopus (Elsevier) or Web of Science (Thomson Reuter).

If the limitations of the methodology used in this study are taken in due account ^12^, the data presented in this paper, as well as in similar papers published in the same period ^4, 13^, can provide useful information to understand and further investigate how the scientific community responds to these global health threats with massive media coverage. For example these findings could be compared to publications trends during or following epidemics such as the Avian Flu or SARS. Forthcoming studies will therefore shed more light on the relationship between media coverage and scientific publications patterns during these major public health events. This information will assist scholars, public health officials and policymakers in their efforts to improve scientific research policies with the goal of maximizing both societal health and knowledge advancement.

## Additional methodological notes

Publication output was determined by using the free and public search engine PubMed. All searches were performed during February 2015, except for the updated searches performed in September 2015 (‘Update September 2015’ sheet, Figure S37). All data and searching details are included in the accompanying database containing ‘sheets 1–9’, the ‘clinical trial term’ sheet, and the ‘Canada’, ‘China’, ‘France’, ‘Germany’, ‘Guinea’, ‘Liberia’, ‘Sierra Leone’, ‘UK’ and ‘USA’ sheets.

## Data availability

The data referenced by this article are under copyright with the following copyright statement: Copyright: © 2015 Ballabeni A and Boggio A

Data associated with the article are available under the terms of the Creative Commons Zero "No rights reserved" data waiver (CC0 1.0 Public domain dedication). 

*Figshare:* Dataset and selected figures of biomedical publications on Ebola in 2014. doi: 10.6084/m9.figshare.1566801 ^14^

## Figures and Tables

**Figure S22.**
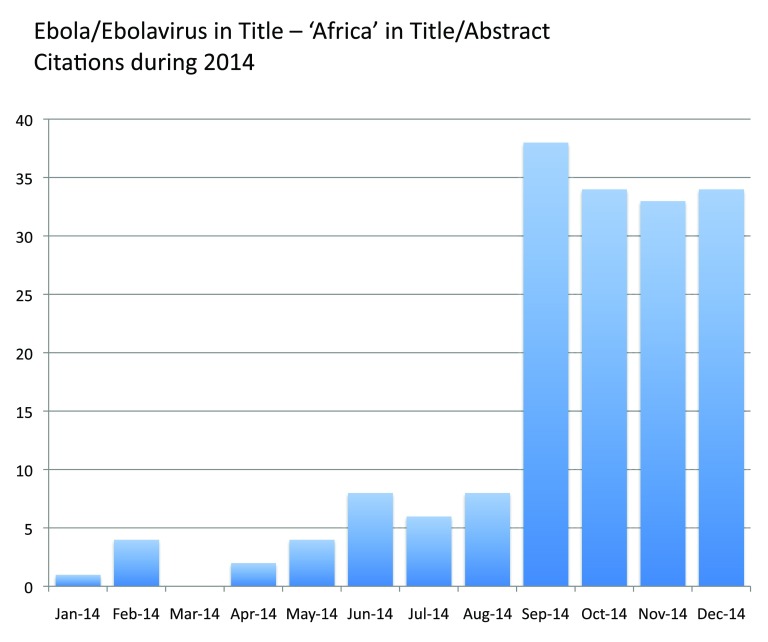
Numbers of citations (‘ebola’ or ‘ebolavirus’ in title) during 2014 with search term ‘ Africa’ in title/abstract.
