# Supplementary material for: Publications in PubMed on Ebola and the 2014 outbreak
Source: F1000Res. 2015 Oct 9;4:68. Originally published 2015 Mar 13. [Version 2] doi: 10.12688/f1000research.6206.2 (PMC4629280; doi:10.12688/f1000research.6206.2)
Supplement: Dataset and selected figures of biomedical publications on Ebola in 2014 — The database contains data about the biomedical publications on Ebola in 2014.The volumes of publications and the classifications were determined by using the PubMed search engine. The data are ordered according to different criteria. Columns/row headings and embedded comments describe the contents of the columns/rows. Columns A-K of sheets ‘Clinical Trial term’, ‘Canada’, ‘China’, ‘France’, ‘Germany’, ‘Guinea’, ‘Liberia’, ‘Sierra Leone’, ‘UK’, ‘USA’ were from csv files downloaded from PubMed searches. Further information about the methodology and the data is contained in the associated article. All data and figures mentioned in the associated article and this figshare entry are linked to a sheet of this database. Figure 3 Numbers of citations with ‘ebola’ or ‘ebolavirus’ in title from 1995 to 2014. Figure 4 Numbers of citations (abstract available) with ‘ebola’ or ‘ebolavirus’ in title from 1995 to 2014. Figure 5 Proportions of citations (‘ebola’ or ‘ebolavirus’ in title) with abstract available from 2005 to 2014. Figure 15 Proportions of citations (‘ebola’ or ‘ebolavirus’ in title) with abstract available with the indicated search terms in the title/abstract. Year 2014. Figure 16 Proportions of citations (‘ebola’ or ‘ebolavirus’ in title) with the search term ‘clinical trial’ in the title/abstract. The number of publications about original clinical trial studies is also indicated. Year 2014. Figure 17 Numbers of citations (‘ebola’ or ‘ebolavirus’ in title) per month. Year 2014. Figure 18 Numbers of citations (‘ebola’ or ‘ebolavirus’ in title) with abstract available per month. Year 2014. Figure 19 Subjective classification for ‘current outbreak’ focus of 2014 citations (‘ebola’ or ‘ebolavirus’ in title). Remaining citations were assigned to one (and only one) of the indicated discipline/area categories. Year 2014. Figure 20 Subjective classification for current outbreak focus or, alternatively, for the indicated discipline/area categories of 2014 citations (‘ebola [file f1000research-4-7680-s0000.tgz › 3571604]

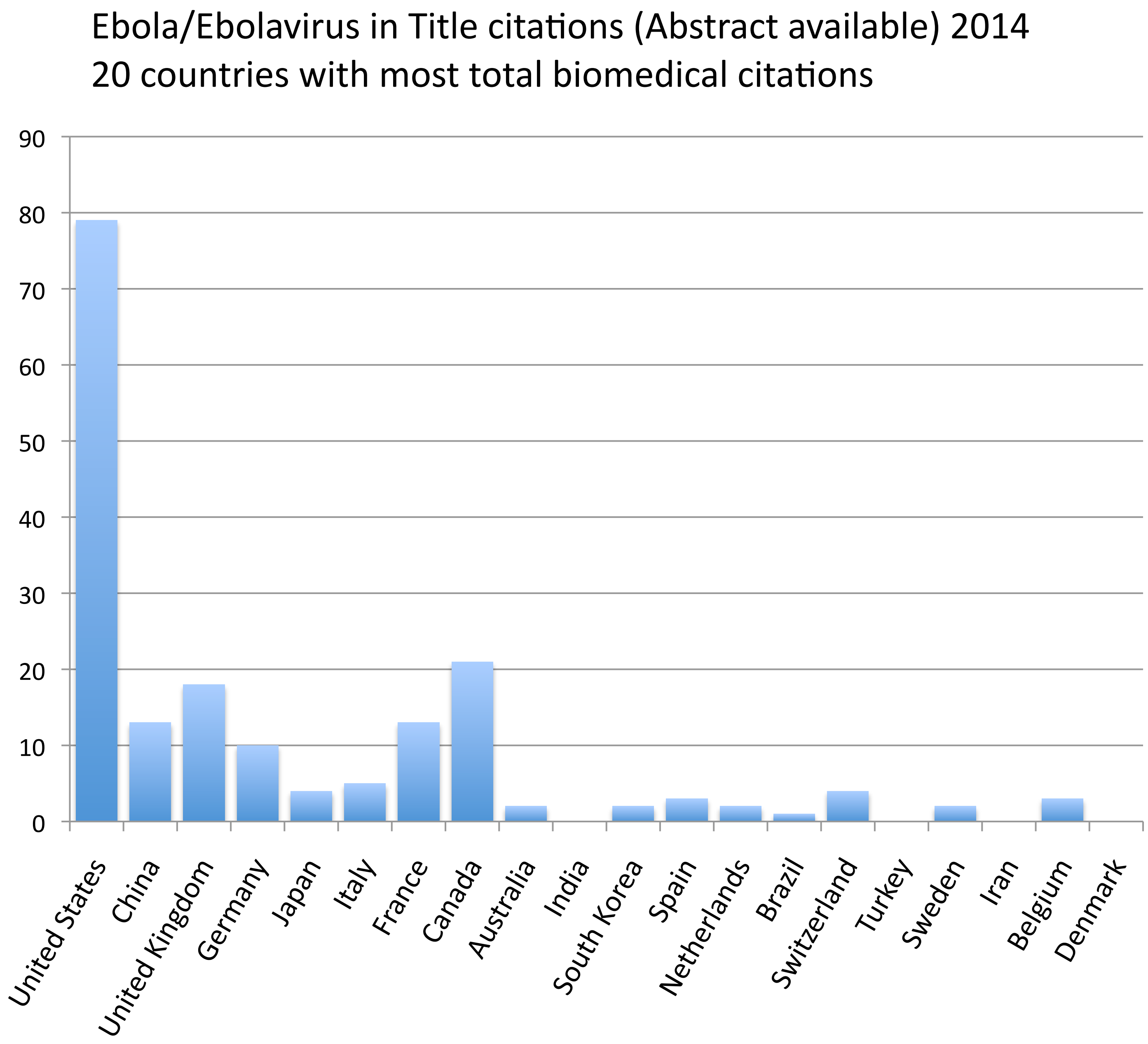
Ebola/Ebolavirus in Title citations (Abstract available) 2014
20 countries with most total biomedical citations
90
80
70
60
50
40
30
20
10
0
United States
China
United Kingdom
Germany
Japan
Italy
France
Canada
Australia
India
South Korea
Spain
Netherlands
Brazil
Switzerland
Turkey
Sweden
Iran
Belgium
Denmark

Fig. 29
