# Supplementary material for: Publications in PubMed on Ebola and the 2014 outbreak
Source: F1000Res. 2015 Oct 9;4:68. Originally published 2015 Mar 13. [Version 2] doi: 10.12688/f1000research.6206.2 (PMC4629280; doi:10.12688/f1000research.6206.2)
Supplement: Peer review history

# Peer review: Referee response for version 2

**DOI:** 10.5256/f1000research.7680.r10739

**Published:** 2016-02-29

**Authors:** Alfonso Rodriguez-Morales

**Affiliations:** 1 Faculty of Health Sciences, Universidad Tecnológica de Pereira, Pereira, Colombia

The authors have properly corrected the manuscript. Now, this article is significantly contributing to the understanding of the pattern of global research on Ebola.

I have read this submission. I believe that I have an appropriate level of expertise to confirm that it is of an acceptable scientific standard.

# Peer review: Referee response for version 2

**DOI:** 10.5256/f1000research.7680.r8946

**Published:** 2015-10-26

**Authors:** Ann Kelly

**Affiliations:** 1 Department of Sociology, Philosophy and Anthropology, University of Exeter, Exeter, UK

The authors have sufficiently responded to my queries; it is an interest paper illuminating not only the interlinkages between media and scientific research, but also the shortfalls in collaboration across the North and South.

I have read this submission. I believe that I have an appropriate level of expertise to confirm that it is of an acceptable scientific standard.

# Peer review: Referee response for version 1

**DOI:** 10.5256/f1000research.6655.r9648

**Published:** 2015-07-27

**Authors:** Ann Kelly

**Affiliations:** 1 Department of Sociology, Philosophy and Anthropology, University of Exeter, Exeter, UK

The sheer volume of Ebola Virus Disease (EVD) scholarship published over the past year is unprecedented in the forty-year history of the disease. This paper provides a close examination of what is reported as an ‘over 12-fold’ increase in biomedical output, scrutinizing trends in publication content, authorship, disciplinary orientations and institutional origins, as well as availability. A central conclusion of this analysis is that EVD-literature surges in late 2014, coinciding with both the peak of the outbreak in West Africa and media attention.

The paper benefits from a robust methodological approach; the search criteria are well considered and rigorously applied. Some further detail about the timelines for publication might have been useful: for instance, how long from submission would a research article take to be published, and whether different timeframes exist for research as opposed to commentary pieces. This further analysis might qualify the claims regarding the simultaneity of the volume of publications and the dramatic rise in Ebola cases.

That being said, the granularity of the sub-analysis—e.g. assessing the number of papers authored by researchers affiliated with institutions in West Africa—generates some intriguing insights into the shifts in scientific interest from basic Ebola science to the specific features of the current outbreak, and suggests some of the constraints of scientific collaborations in countries with weak research infrastructure. The subject analysis also offers some tantalizing leads into the prominence of certain research areas—for instance vaccines—during a time a global health crisis.

My central reservation is that these connections are not explored further. The general point that media coverage and research agendas are inter-related seems hardly surprising. Indeed, scholarship in scientific communication and science and technology studies has illuminated these links as well as the politics of funding in considerable detail. With such a potentially rich dataset to hand, it is frustrating that the authors are not more ambitious in their interpretations, comparing for instance, a similar set of trends in the literature published during the Avian Flu or SARS epidemics. Has the emphasis on ‘preparedness’ shifted? In what ways has the institutional geography of authorship changed?

In general, this is an intriguing article that offers a solid overview on the dynamics of scientific output in response to a global threat. My only regret is that the analytical agenda was not bolder, for it could have led to a few more surprising conclusions.

I have read this submission. I believe that I have an appropriate level of expertise to confirm that it is of an acceptable scientific standard, however I have significant reservations, as outlined above.

# Response

**Published:** 2015-09-21

**Authors:** Andrea Ballabeni

**Affiliations:** Harvard School of Public Health, USA

We would like to thank Dr Ann Kelly for her review. We are glad for her appreciation of the paper and want to address her two concerns.

The comment “ *how long from submission would a research article take to be published, and whether different timeframes exist for research as opposed to commentary pieces*” is a very good one. Indeed we agree with the referee that these timeframes must be taken in consideration and added a short discussion in the Conclusions section to address this concerns. However, we think that an in-depth quantitative analysis of this topic is complex and not suitable for the purposes and length-constraints of this short “research note.” Future studies will certainly shed more light on these timeframes.

With regard to the comment: “ *scholarship in scientific communication and science and technology studies has illuminated these links as well as the politics of funding in considerable detail. With such a potentially rich dataset to hand, it is frustrating that the authors are not more ambitious in their interpretations, comparing for instance, a similar set of trends in the literature published during the Avian Flu or SARS epidemics. Has the emphasis on ‘preparedness’ shifted? In what ways has the institutional geography of authorship changed?*”

We agree with the referee that it would be very interesting to perform these additional investigations. For this reason we have added a short discussion in the Conclusions section. However, though potentially informative, these additional analyses were beyond the purposes and the length-constraints of this short research note. Even if we certainly agree that it will be worth performing these investigations in future studies, we hope that the referee concurs that the lack of these further analyses does not diminish the quality of the data presented in this research note. In fact the scope of the “research note” was to provide a set of good data to be used in and to stimulate further and broader studies.

We hope that, based on this clarification, the referee can now fully approve our article.

Moreover, we have made a few additional changes (summarized in Amendments from Version 1 box) that hopefully will be appreciated by the referee.

We would like to thank again Dr Kelly for her thoughtful and helpful comments.

Best regards,

Andrea Ballabeni and Andrea Boggio

# Peer review: Referee response for version 1

**DOI:** 10.5256/f1000research.6655.r7968

**Published:** 2015-03-17

**Authors:** Alfonso Rodriguez-Morales

**Affiliations:** 1 Faculty of Health Sciences, Universidad Tecnológica de Pereira, Pereira, Colombia

Very interesting article.

General comments:

- The appropriateness of the title: Ok.
- Whether the abstract provides an adequate summary of the article: I would include the total number of articles found in the abstract.
- If there is a comprehensive explanation of study design, methods and analysis, and their suitability to the investigation: Ok.
- Whether the conclusions are balanced and justified on the basis of the results: I suggest commenting more on the implications of the findings in regard the advance to obtain an available vaccine for Ebola.
- With regards to the data (if applicable), whether sufficient information has been provided for replication of the experiment, and/or if the data are in a usable format: Ok.

I have only two recommendations:

1. To further discuss why the authors only assessed the database PubMED/Medline, and why Science Citation Index and Scopus were not included. Even we can consider to include also some African database.
2. V1 of this article appears online on March 13, 2015, however, 7 days before, on March 6, 2015, appeared online the following article:
  Cruz-Calderón S, Nasner-Posso KM, Alfaro-Toloza P, Paniz-Mondolfi AE, Rodríguez-Morales AJ. **A bibliometric analysis of global Ebola research**. *Travel Medicine & Infectious Disease *2015 Epub Ahead Mar 6; available online at: http://www.sciencedirect.com/science/article/pii/S1477893915000344. doi: 10.1016/j.tmaid.2015.02.007.
  Then, I consider this should be cited, as a preliminary approach to this topic. Also comparing the findings between both. This analysis included PubMED/Medline, Science Citation Index and Scopus.

I have read this submission. I believe that I have an appropriate level of expertise to confirm that it is of an acceptable scientific standard.

# Response

**Published:** 2015-10-05

**Authors:** Andrea Ballabeni

**Affiliations:** Harvard School of Public Health, USA

We would like to thank Dr Alfonso Rodriguez-Morales for his review. We are glad the referee likes and approves our article. Here are our responses to his comments/suggestion.

As suggested by the referee, we have now included the number of total publications in the abstract, based on an updated search performed in September 2015.

With reference to the implication of the findings in regard to the advances to obtain a vaccine, we think this is a more complex topic that is beyond the purpose of this short research note. However, we certainly agree with the referee that this is an important issue that would be worth investigating/discussing in future studies and opinion pieces.

With regard to the two recommendations:

1. We think that given the comprehensiveness and breadth of the PubMed-searched databases, the results presented in this paper are a bona fide representation of the literature. In any case, even if this is beyond the scope of this short research note, we agree with the referee that it could be useful to confirm these data by using also other databases such as the Web of Science (Thomson Reuter) and the Scopus (Elsevier). To address this, we have added a few comments in the Conclusions section. Moreover, to emphasize that the data presented in this paper are based on PubMed-searches we have modified the title.Moreover, we believe that the fact that this paper is based on a search engine/databases that is free and easily accessible adds value to the results.
2. We have added the reference suggested and authored by the referee. We think that this referenced paper, published a few days before our paper, indeed provides interesting information that supports and complements the data of our study. We have also added a short comment in the Conclusions section. 

Moreover, we have made a few additional changes (summarized in Amendments from Version 1 box) that hopefully will be appreciated by the referee.

We would like to thank again Dr Rodriguez-Morales for his advice.

Best regards,

Andrea Ballabeni and Andrea Boggio
